# Supplementary material for: Monoubiquitination of ASXLs controls the deubiquitinase activity of the tumor suppressor BAP1
Source: Nat Commun. 2018 Oct 22;9:4385. doi: 10.1038/s41467-018-06854-2 (PMC6197237; doi:10.1038/s41467-018-06854-2)
Supplement: Supplementary file 1 — Supplementary Information [file 41467_2018_6854_MOESM1_ESM.pdf]

Supplementary Information

Monoubiquitination of ASXLs controls the deubiquitinase activity of the tumor suppressor BAP1

Daou et al.

a

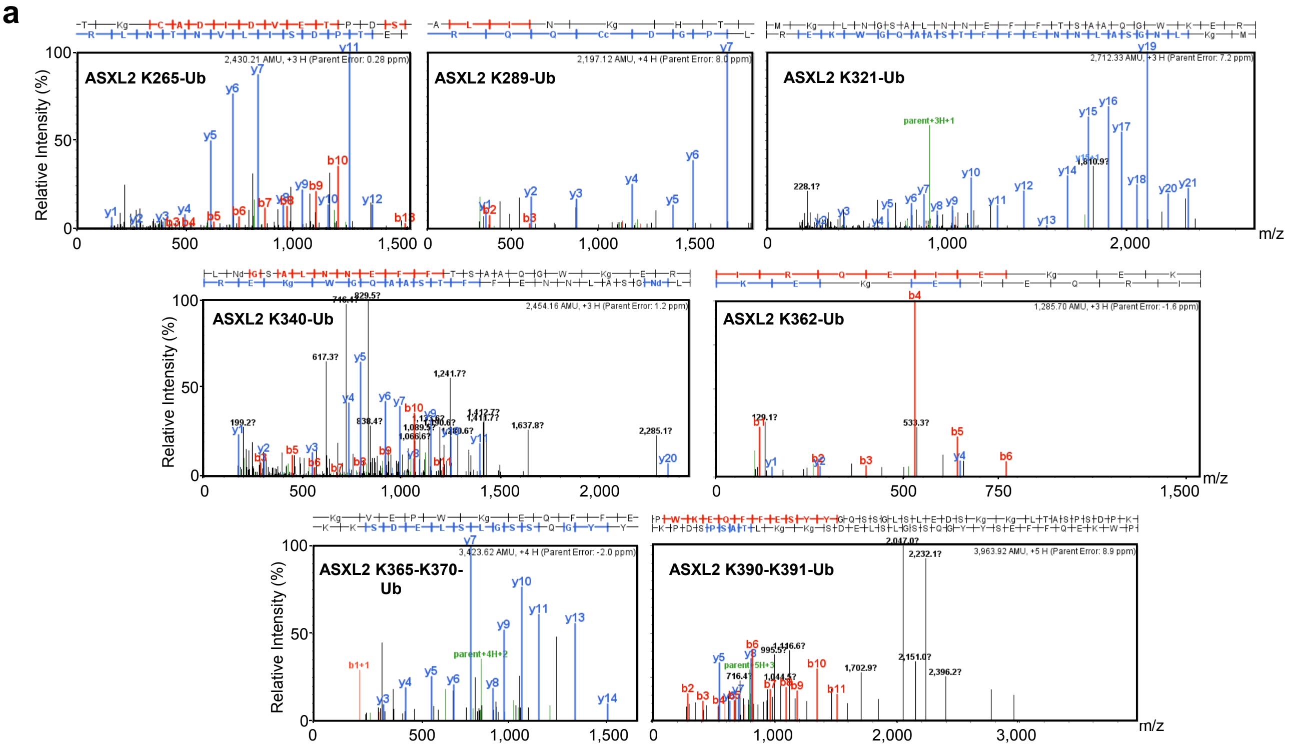

b

Proteins	Ub Sites	This study	PhosphoSitePlus records (https://www.phosphosite.org/)
ASXL2	K265	TKCADIDVETPDSILV NTNLR	
ASXL2	K289	ALINKHTFSVLPGDCQ QR	
ASXL2	K321	MKLNGSALNNEFFTS AAQGWKER	
ASXL2	K340	LNGSAMNNEFFTSAA QGWKER	
ASXL2	K362	IRQEIEKEK	
ASXL2	K365 K370	KVEPWKEQFFESYYG QSSGLSLEDSKK	24 104
ASXL2	K390 K391	PWKEQFFESYYGQSS GLSLEDSKKLTASPS DPK	
ASXL2	K1245		3
ASXL2	K1259		2
ASXL1	K351	KKVEQWKEKF	55
ASXL1	K353		1
ASXL1	K362		3
ASXL1	K367		2
ASXL1	K1371		1
ASXL1	K1379		2
ASXL1	K1419		2
ASXL1	K1437	GLSEPLEPSSLPSQLS IKQAFYGKL	3
ASXL1	K1446		1
ASXL3	K350	EKKTEPWKEKFFERF	2
ASXL3	K352		1

c

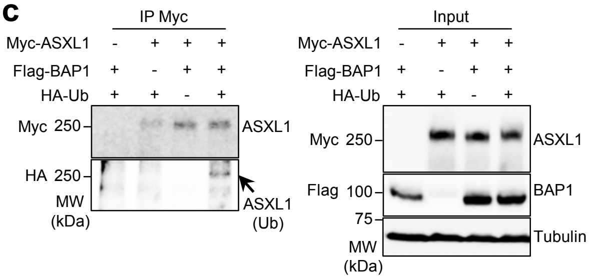

d

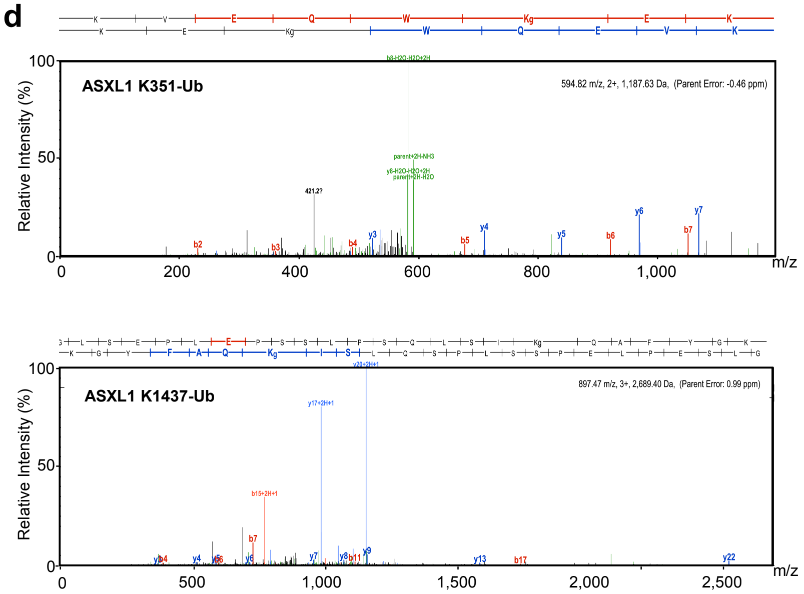

e

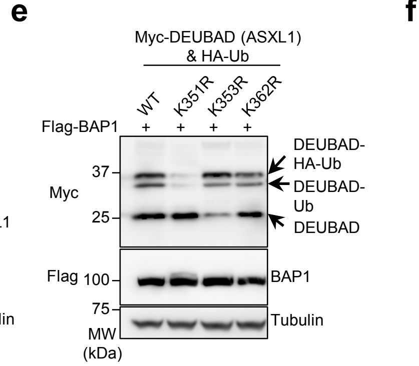

f

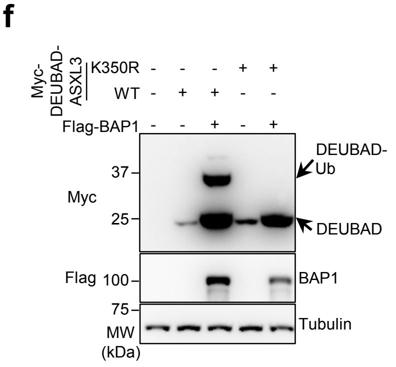

Supplementary Figure 1

Supplementary Figure 1. Characterization of DEUBAD monoubiquitination.

a Identification of ubiquitination sites in ASXL2. HEK293T cells were transfected with Flag-ASXL2 expression vector. Flag purified ASXL2 from total extracts was analyzed by mass spectrometry (MS). MS/MS spectra of ASXL2 ubiquitinated lysine residues are indicated. The modified lysine residues by the C-terminal Gly-Gly fragment of ubiquitin are indicated as Kg. n=1 biological sample. **b** Summary of ASXL1, ASXL2 and ASXL3 ubiquitination sites identified in this study and others. Number of records of the corresponding ubiquitination sites were extracted from PhosphoSitePlus. **c** Myc-ASXL1 was co-transfected in HEK293T cells with and without Flag-BAP1 or HA-Ub and immunoprecipitation with Myc antibody was performed to analyze ASXL1 ubiquitination as indicated. n=3 independent biological replicates. **d** Identification of ubiquitination sites in ASXL1. HEK293T cells were transfected with expression vector for Flag-ASXL1. ASXL1 was purified for MS analysis. MS/MS spectra indicating Ub remnant on K351 and K1437. The modified lysine residues within the peptide were indicated as described above. n=1 biological sample. **e** Lysine 351 is the BAP1-dependent monoubiquitination site of ASXL1. HEK293T cells were transfected as indicated with Myc-DEUBAD constructs for ASXL1 and cell lysates were used for western blotting. n=3 independent biological replicates. **f** ASXL3 is ubiquitinated on its DEUBAD on K350 in BAP1-dependent manner. HEK293T cells were transfected with Myc-DEUBAD (ASXL3) or Myc-DEUBAD (ASXL3) K350R with or without Flag-BAP1 and DEUBAD monoubiquitination was analyzed as indicated. Note that ASXL3 DEUBAD is highly stabilized in the presence of BAP1. n=2 biological sample. Tubulin was used as a loading control for panels **c, e** and **f**.

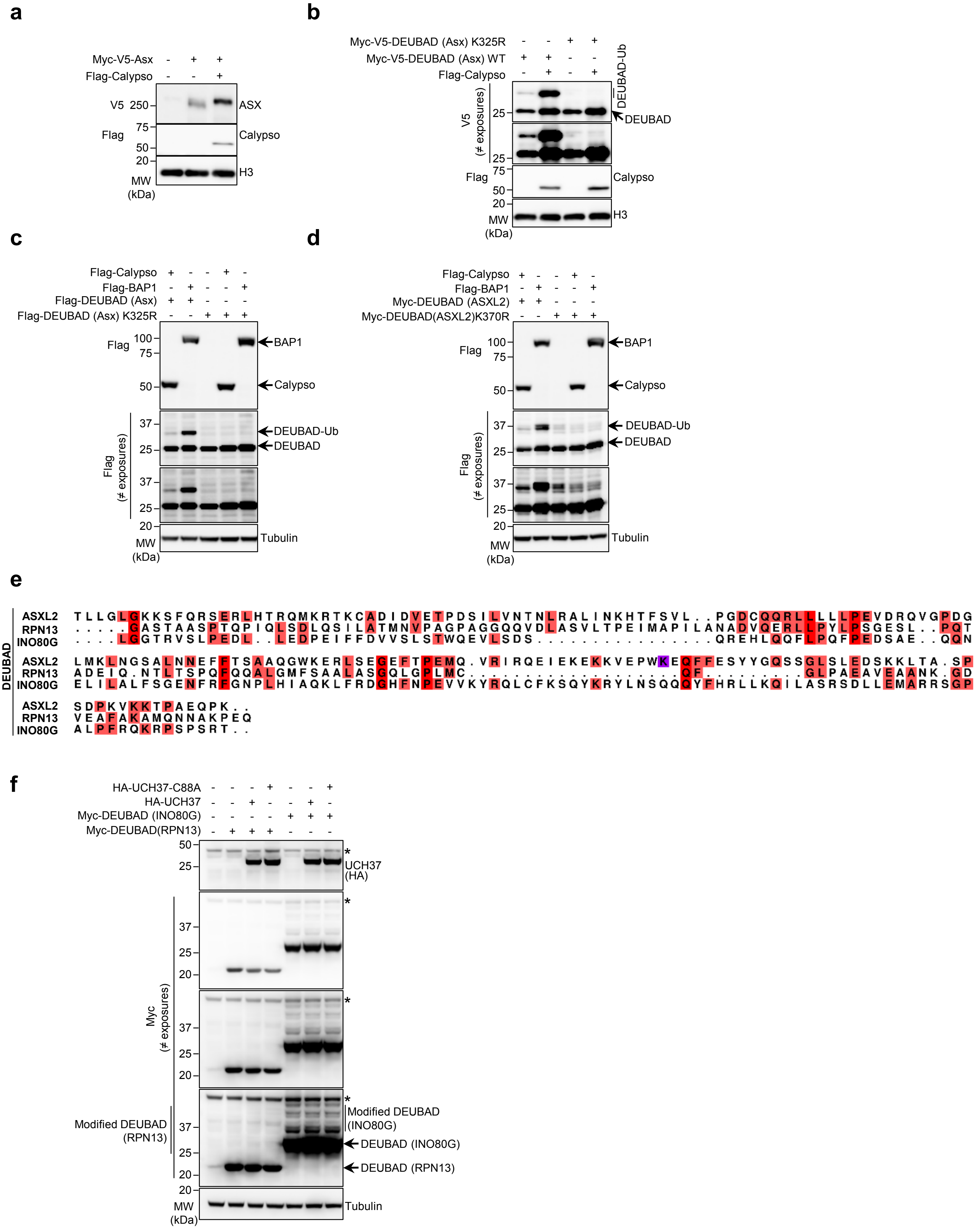

Supplementary Figure 2

Supplementary Figure 2. Monoubiquitination of DEUBAD is conserved in *Drosophila*.

a *Drosophila* Asx is monoubiquitinated in Calypso-dependent-manner. Myc-V5-Asx was transfected with and without Flag-Calypso in S2 cells. Note the Asx band upper shift suggestive of its modification in the presence of Calypso. n=3 biological replicates. **b** *Drosophila* DEUBAD K325 is monoubiquitinated in Calypso-dependent-manner. Myc-V5-DEUBAD (Asx) and its K325R mutant constructs were co-transfected with and without Calypso expression constructs in S2 cells. The cells were then harvested for western blotting analysis. n=3 biological replicates. **c** *Drosophila* DEUBAD is ubiquitinated in human cells**.** HEK293T cells were transfected with either Flag-BAP1 or Flag-Calypso along with Flag-DEUBAD (Asx) or Flag-DEUBAD (Asx) K325R and harvested for immunoblotting analysis as indicated. n=2 biological replicates. **d** Calypso promotes DEUBAD ubiquitination in human cells. HEK293T cells were transfected with either Flag-BAP1 or Flag-Calypso along with Myc-DEUBAD (ASXL2) or Myc-DEUBAD (ASXL2) K370R and harvested for immunoblotting analysis as indicated. n=2 biological replicates. **e** K370 of ASXL2 DEUBAD is not conserved in RPN13 or INO80G DEUBADs. Multiple sequence alignment of the DEUBAD domains of ASXL2, RPN13 and INO80G. The K370 of ASXL2, including its peptide sequence context, is not found in RPN13 or INO80G DEUBADs. The K370 is highlighted in purple. **f** UCH37 does not promotes RPN13 or INO80G DEUBAD monoubiquitination. HEK293T cells were transfected with either Myc-DEUBAD (RPN13) or Myc-DEUBAD (INO80G) with and without Flag-UCH37 or its catalytic dead mutant (C88A). Cells were harvested for immunoblotting analysis as indicated. The star indicates a non-specific band. n=3 biological replicates. Tubulin was used as a loading control for panels **c, d** and **f**. Histone H3 was used as a loading control for panels **a, b**.

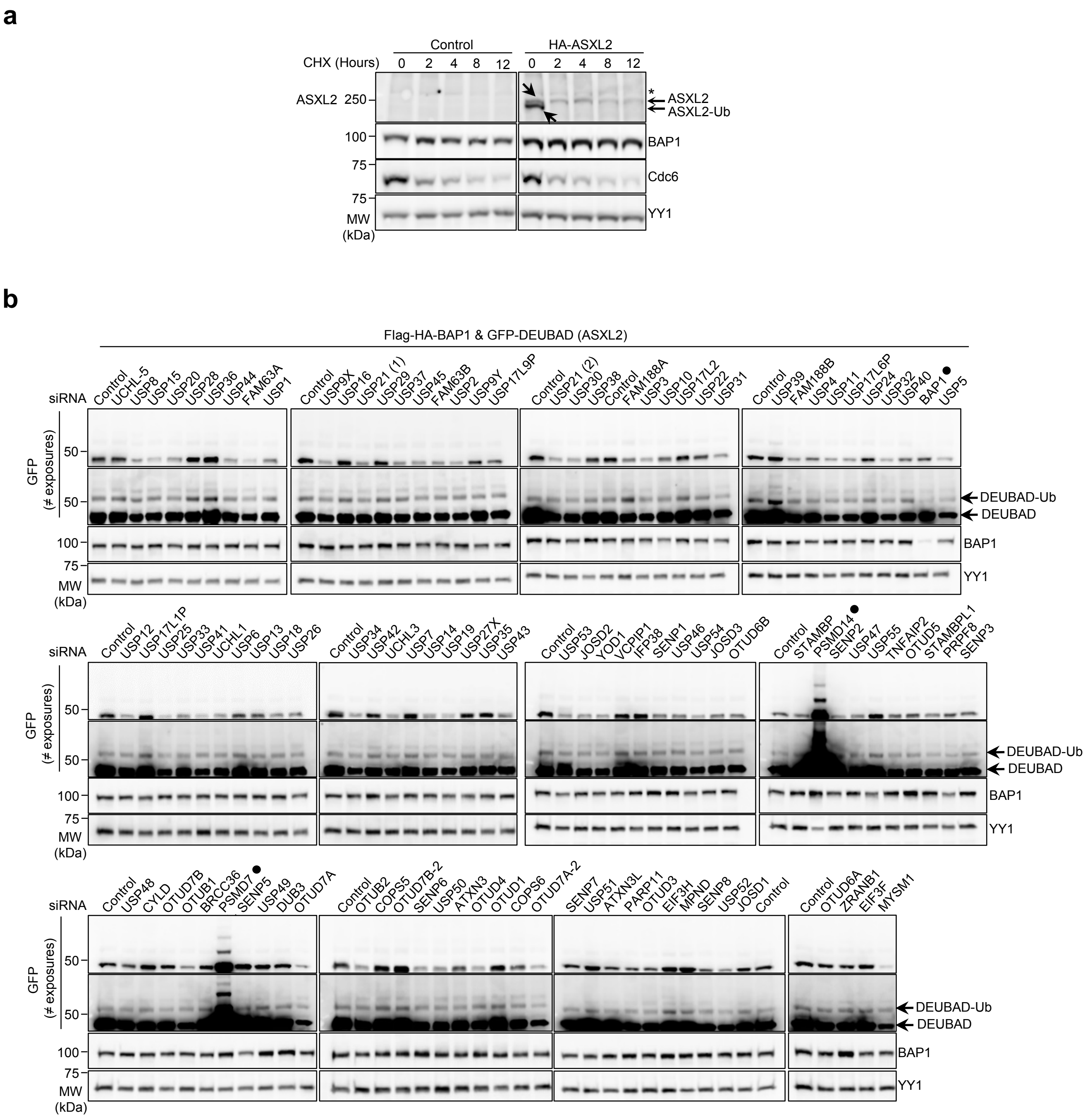

Supplementary Figure 3

Supplementary Figure 3. ASXL2 stability is regulated by the proteasome and deubiquitination.

a Monoubiquitinated ASXL2 is very stable comparatively to the unmodified form. U-2 OS cells stably expressing control empty vector or HA-ASXL2 were treated with cycloheximide (CHX) for the indicated times and harvested for immunoblotting analysis. The arrows indicate the non-modified and the monoubiquitinated form of ASXL2. The star indicates a non-specific band. n=2 biological replicates. **b** DUB siRNA screen identifies potential regulators of DEUBAD ubiquitination. U-2 OS cells stably expressing Flag-HA-BAP1 and GFP-DEUBAD (ASXL2) were transfected with an siRNA library for human deubiquitinases assembled from individual siRNAs provided by Sigma (see **Supplementary Table 1**). Cell extracts were used for immunoblotting. The black dots indicate the siRNA for BAP1, PSMD14 and PSMD7. n=1 biological replicate. YY1 was used as a loading control for panels **a** and **b**.

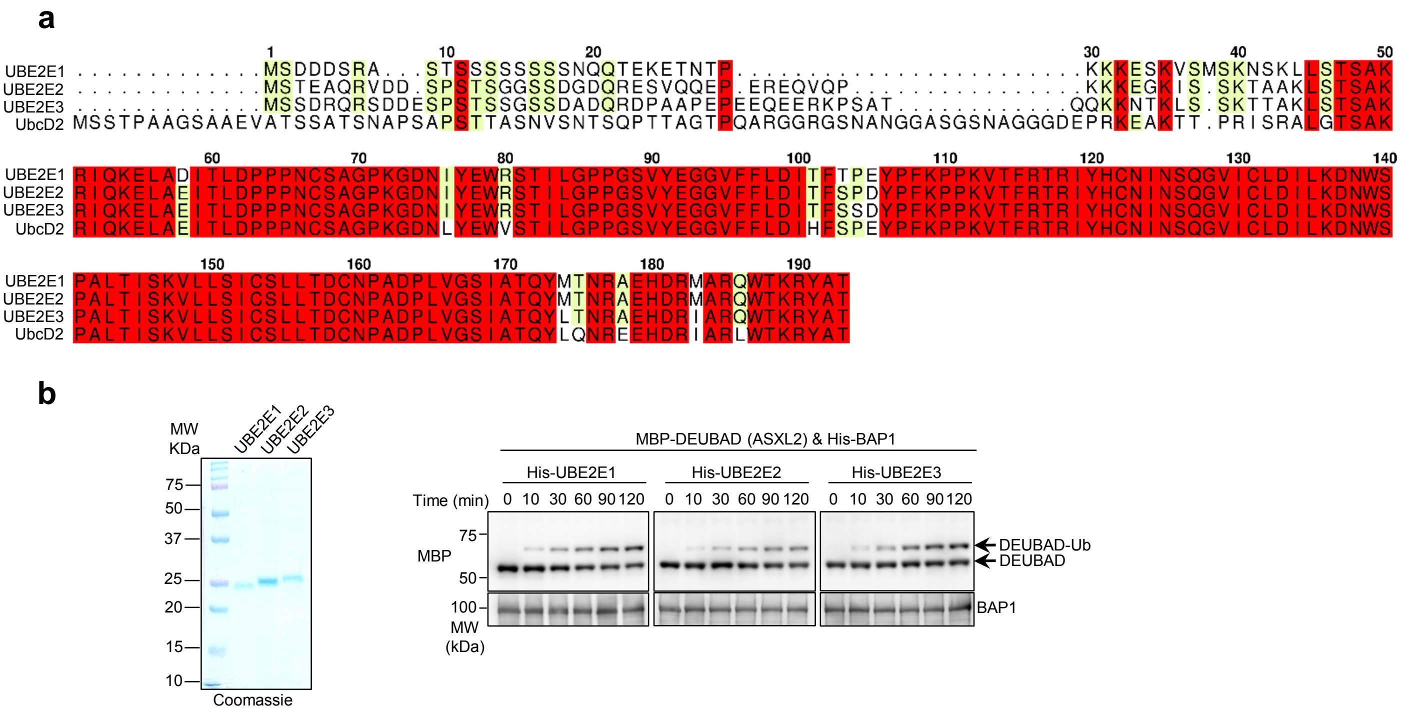

Supplementary Figure 4

Supplementary Figure 4. UBE2E Ub-conjugating enzymes catalyze DEUBAD monoubiquitination.

a Alignment of human UBE2E1, UBE2E2 and UBE2E3 sequences with *Drosophila* UbcD2. **b** UBE2E1, UBE2E2 and UBE2E3 catalyze DEUBAD ubiquitination *in vitro***.** Left panel, analysis of UBE2E1, UBE2E2 and UBE2E3 proteins using coomassie blue staining. His-tagged human UBE2E2 and UBE2E3 proteins were purified from bacteria and recombinant His-UBE2E1 was from Boston Biochem. Right panel**,** bacteria purified His-BAP1/MBP-DEUBAD (ASXL2) complex was incubated with recombinant His-tagged UBE2E1 or UBE2E2 or UBE2E3 enzymes for *in vitro* ubiquitination assays for the indicated times. The reactions were stopped at the indicated time points and analyzed by immunoblotting. n=2 biological replicates.

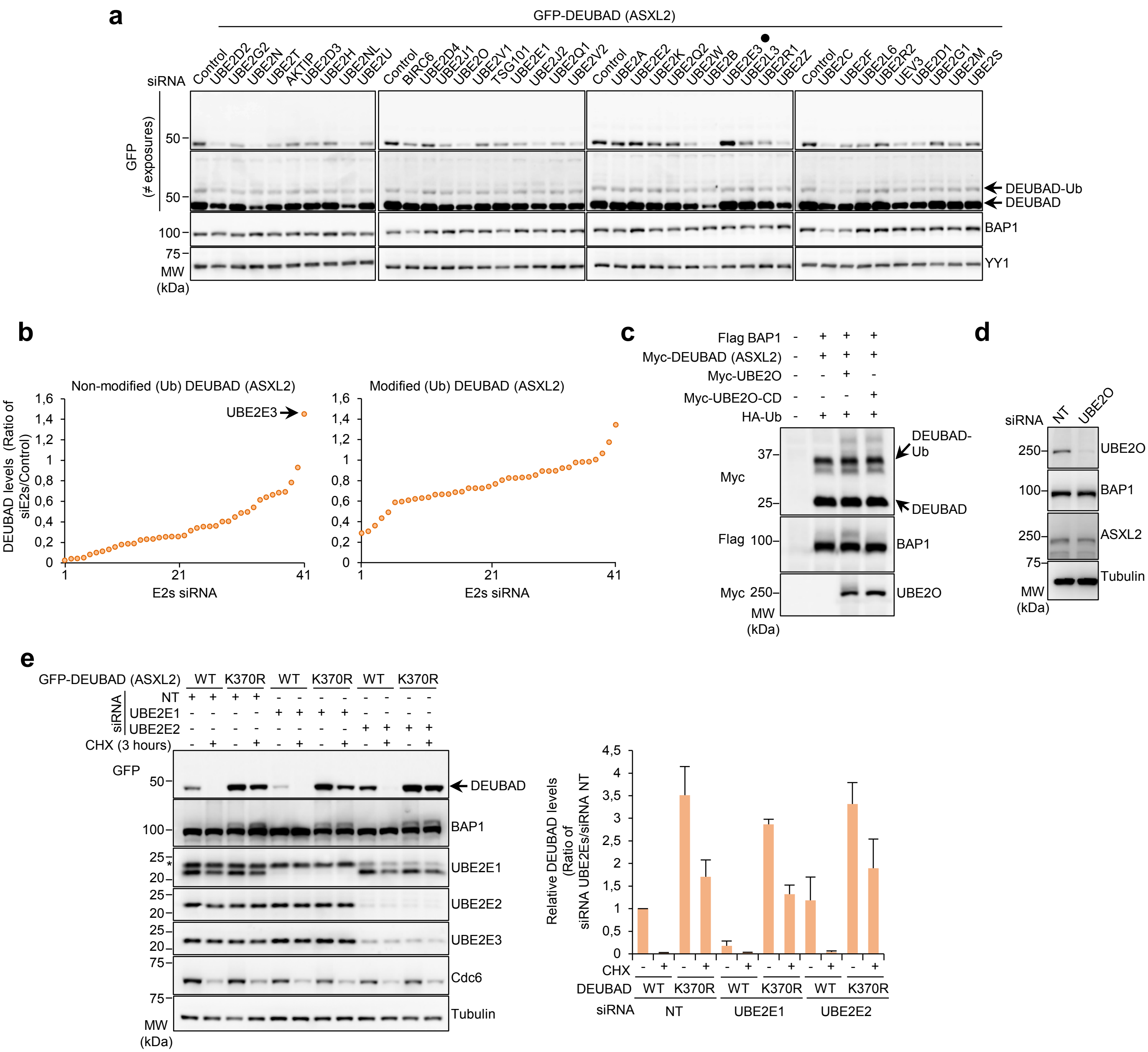

Supplementary Figure 5

Supplementary Figure 5. UBE2E3 regulates DEUBAD stability.

a E2 ubiquitin-conjugating siRNA screen identifies potential regulators of DEUBAD ubiquitination. U-2 OS cells stably expressing GFP-DEUBAD (ASXL2) were transfected with siRNA library for all human E2s and DEUBAD protein levels were analyzed by western blotting. n=1 biological replicate. **b** UBE2E3 is the only E2 whose depletion significantly increases the levels of the non-modified form of DEUBAD (ASXL2). Densitometry analysis of the indicated protein bands in E2s siRNAs versus siRNA control. **c** UBE2O overexpression does not affect DEUBAD ubiquitination. HEK293T cells were transfected with expression vectors for Myc-DEUBAD (ASXL2), Flag-BAP1 and HA-Ub with or without Myc-UBE2O or Myc-UBE2O catalytic dead (CD). Cells were harvested for immunoblotting analysis. n=2 biological replicates. **d** UBE2O depletion does not affect ASXL2 stability. U-2 OS cells were transfected with Non-target (NT) control siRNA as well as UBE2O siRNA and were harvested for immunoblotting analysis as indicated. n=3 biological replicates. **e** Effects of UBE2E1 or UBE2E2 depletion on DEUBAD stability. U-2 OS cells stably expressing either GFP-DEUBAD (ASXL2) or GFP-DEUBAD (ASXL2) K370R were transfected with Non-target siRNA control or siRNA for UBE2E1 or UBE2E2 and then treated with CHX for the indicated times. Cell lysates were used for immunoblotting. Densitometry analysis of DEUBAD protein levels was conducted and presented for each siRNA condition. The star indicates UBE2E2 band detected with UBE2E1 antibody. Error bars represent **s.d** (mean ± SD). n=3 biological replicates. YY1 was used as a loading control for panel **a**. Tubulin was used as a loading control for panels **d** and **e**.

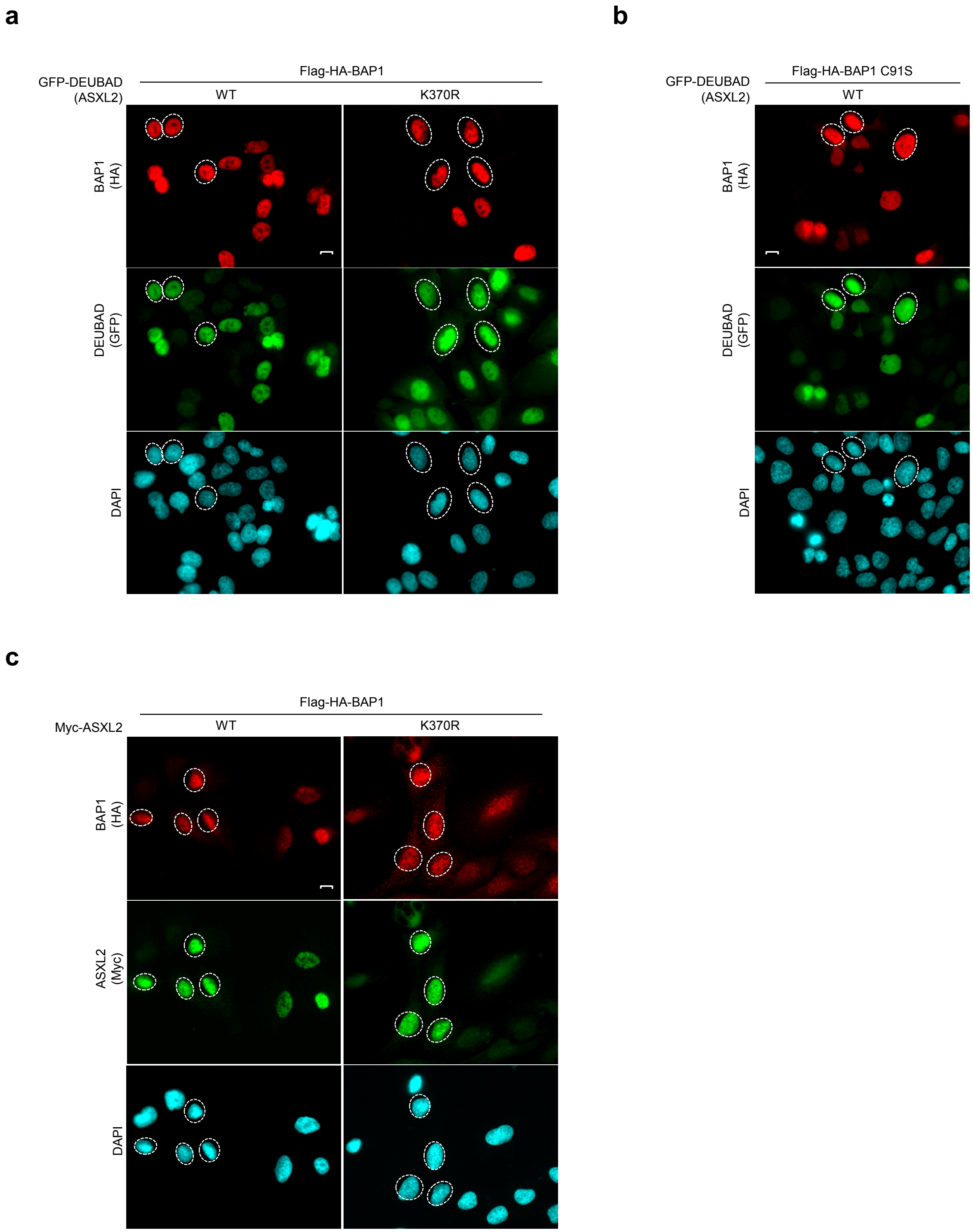

Supplementary Figure 6

Supplementary Figure 6. Expression levels of DEUBAD (ASXL2) and ASXL2 or their corresponding mutants in the presence of BAP1.

a-c U-2 OS cells stably expressing Flag-HA-BAP1 or Flag-HA-BAP1 C91S were transduced with lentivirus expressing constructs for either GFP-DEUBAD (ASXL2) or GFP-DEUBAD (ASXL2) K370R (**a**, **b**) or Myc-ASXL2 WT or Myc-ASXL2 K370R (**c**) and harvested for immunostaining analysis. The encircled cells co-express the different forms of BAP1 with the corresponding forms of ASXL2 proteins. GFP DEUBAD (ASXL2) (green), Myc-ASXL2 (green), HA-BAP1 (red), DAPI (blue). Note that DEUBAD or ASXL2 and BAP1 are generally co-stabilized when co-expressed together. Scale bars: 15 μm. n=3 biological replicates.

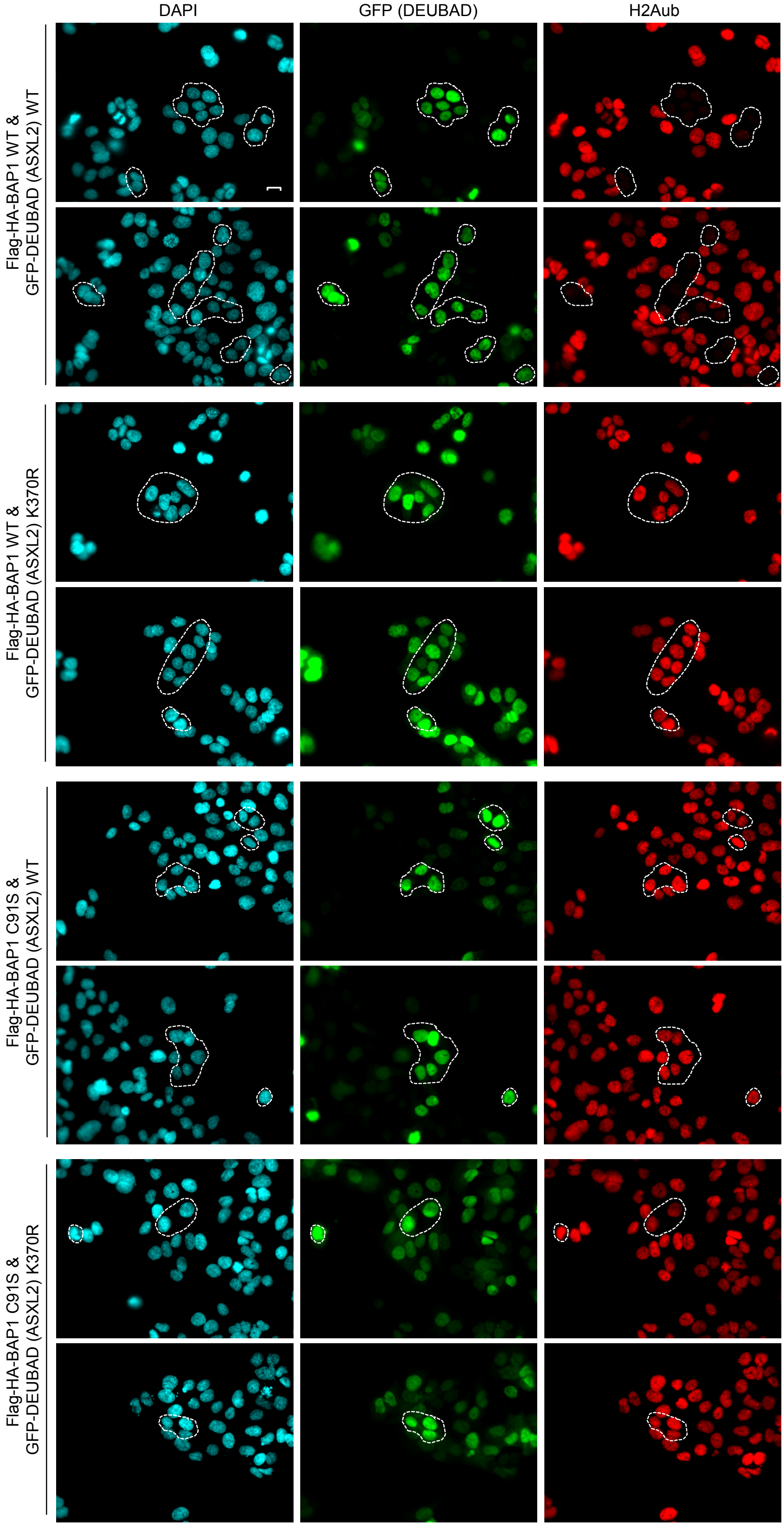

Supplementary Figure 7

Supplementary Figure 7. DEUBAD monoubiquitination promotes BAP1 DUB activity

U-2 OS cells stably expressing Flag-HA-BAP1 or Flag-HA-BAP1 C91S were transduced with lentivirus expressing constructs for either GFP-DEUBAD (ASXL2) or GFP-DEUBAD (ASXL2) K370R and harvested for H2Aub levels analysis by immunostaining. GFP DEUBAD (ASXL2) (green), GFP DEUBAD (ASXL2) K370R (green), H2Aub (red), DAPI (blue). Selected cells showing either decrease or no change of H2Aub levels were encircled. Scale bars: 20 μm. n=3 biological replicates.

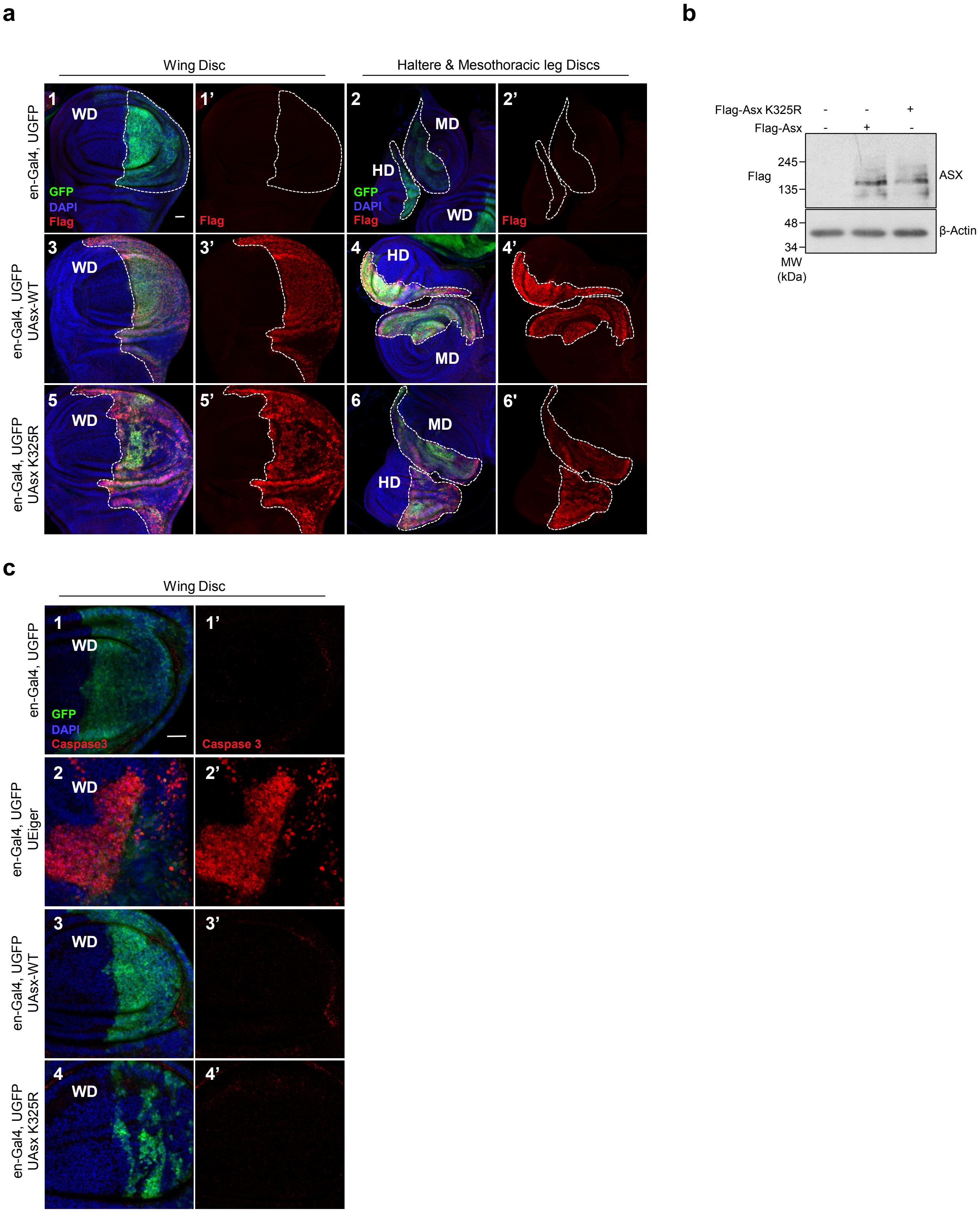

Supplementary Figure 8

Supplementary Figure 8. *Drosophila* Asx WT or K325R are expressed at similar levels in Drosophila tissues

a Immunostaining showing the expression levels of Asx WT or Asx K325R (Flag) during larval disc development. Imaginal discs were stained with anti-Flag (red) for Asx expression and DAPI (blue) for nuclei. GFP staining indicates the expression pattern of *en-Gal4.* The genotypes are indicated*.* WD: Wing imaginal disc, HD: Haltere imaginal disc; MD: Mesothoracic leg imaginal disc. Scale bars: 20 μm. n=3 biological replicates**. b** Immunoblot showing the expression levels of Asx WT or Asx K325R (Flag) in whole larva extracts. Ubiquitous expression was achieved through the use of the ubiquitin-Gal4 driver. Actin was used as a loading control. **c** Overexpression of Asx (WT or K325R) does not induce apoptosis during wing development. Apoptotic cells were stained for cleaved caspase-3. Expression of Eiger was conducted as a positive control for cleaved caspase-3 staining. The genotypes are indicated. GFP and DAPI staining were performed as indicated above. Scale bars: 20 μm. n=2 biological replicates.

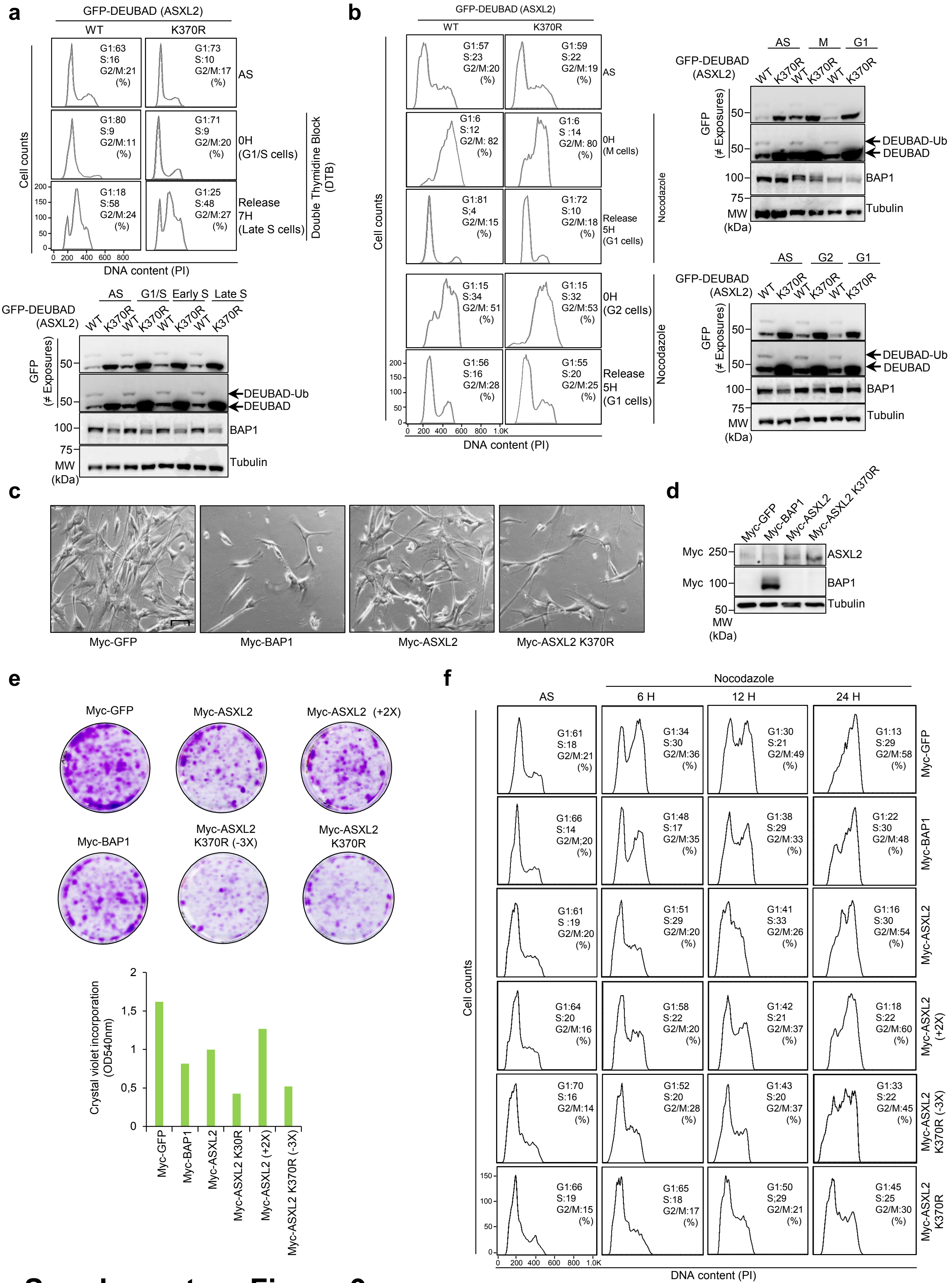

Supplementary Figure 9

Supplementary Figure 9. Expression of ASXL2 K370R reduces mammalian cell proliferation

a-b DEUBAD of ASXL2 is constitutively monoubiquitinated during cell cycle progression. U-2 OS cells stably expressing Flag-HA-BAP1 were transduced with either GFP-DEUBAD (ASXL2) or GFP-DEUBAD (ASXL2) K370R and synchronized in different phases of the cell cycle. Cells were arrested in G1/S by double thymidine block then released for 7 hours and cell cycle progression was analyzed by FACS (**a**). Cells were also treated with nocodazole to induce M arrest. Cells were harvested at the indicated times post-release for FACS analysis (**b**). DEUBAD monoubiquitination levels and BAP1 protein levels were evaluated by immunoblotting. n=2 biological replicates. **c-d** Human primary fibroblasts (LF1) were transduced with lentiviral control or BAP1 or ASXL2 or ASXL2 K370R lentiviral expression vectors. Equal numbers of cells were either plated and phase contrast pictures were taken **(c)** or harvested for protein expression analysis **(d)**. n=3 biological replicates. **e-f** 3T3L1 mouse preadipocytes cells were transduced with different amounts of BAP1, ASXL2 WT or K370R lentivirus particles and equal numbers of cells were either plated for crystal violet staining **(e)** or treated with nocodazole and collected for FACS analysis as indicated **(f).** Note that (+2X) refers to transduction of cells with twice the amount of viruses culture media we normally use for Myc-ASXL2, and (-3X) refers to transduction of the cells with three times less the amount of viruses culture media we normally use for ASXL2 K370R. This adjustment was conducted to correct for the expression levels usually higher for ASXL2 K370R. As: Asynchronous cell population. n=2 biological replicates. Tubulin was used as a loading control for panels **a, b** and **d**.

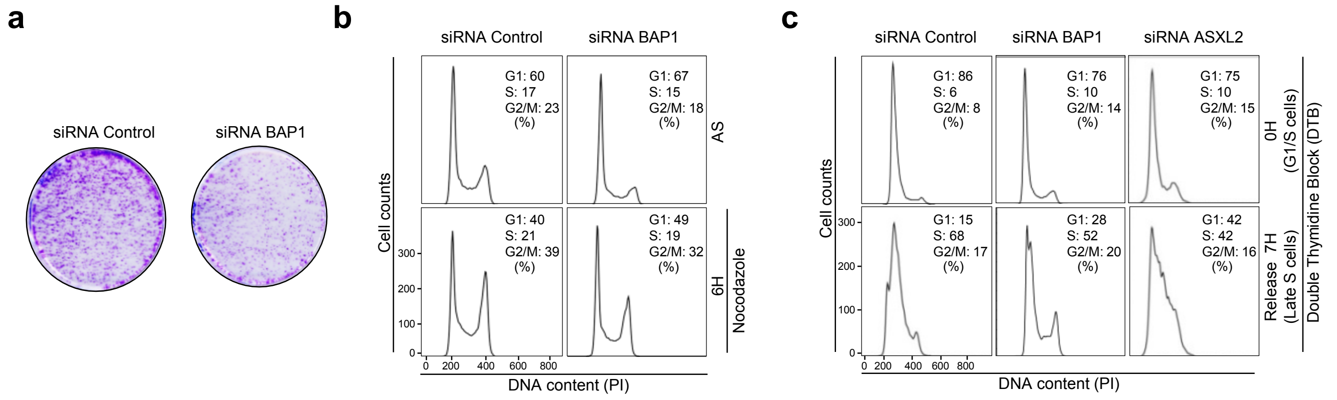

Supplementary Figure 10

Supplementary Figure 10. BAP1 depletion reduces cell proliferation.

a-c U-2 OS cells were transfected with NT siRNA control or siRNA for BAP1. Equal number of cells were plated for colony formation assay (**a**) or treated with nocodazole for FACS analysis (**b**). Equal number of U-2 OS cells transfected with NT siRNA control or siRNA for BAP1 or ASXL2 were subjected to double thymidine block as indicated, and cell cycle progression was analyzed by FACS following release from S-phase block (**c**). n=3 biological replicates.

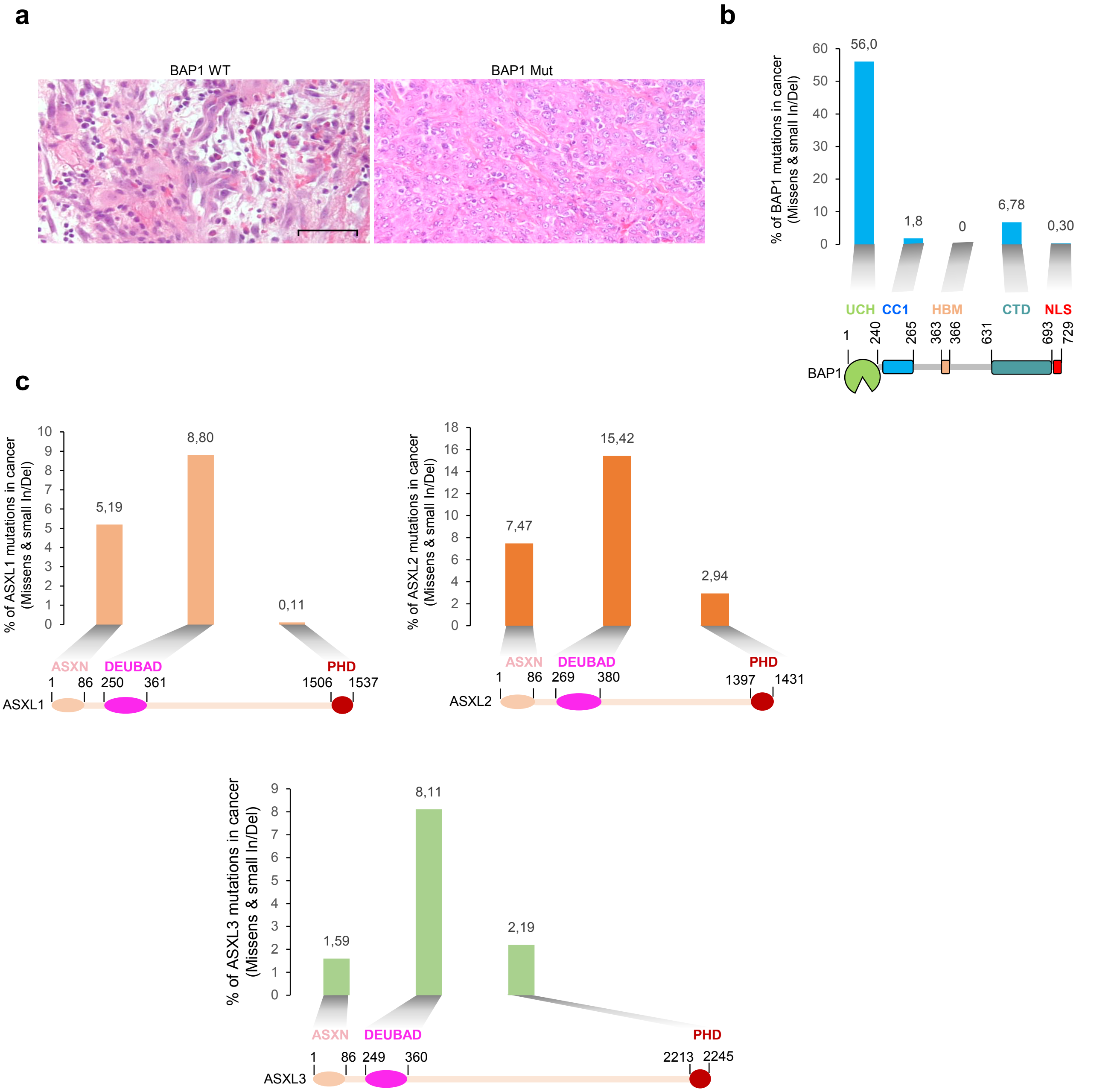

Supplementary Figure 11

Supplementary Figure 11. Cancer-associated mutations of BAP1 and ASXLs.

a Haemotoxylin and Eosin staining of mesothelioma biopsies corresponding to **Figure 9j**. Images were taken at 100X magnification. Scale bars: 100 μm. **b-c** Schematic representation of BAP1 (**b**) and ASXLs (**c**) showing cancer mutations frequencies among their functional domains. Data integrating multiple cancer types was compiled from C-Bioportal database. Note that only missense mutations and small in-frame insertions/deletions were considered.

Supplementary Figure 12. Uncropped immunoblots used for the indicated proteins in the **Figure 1**

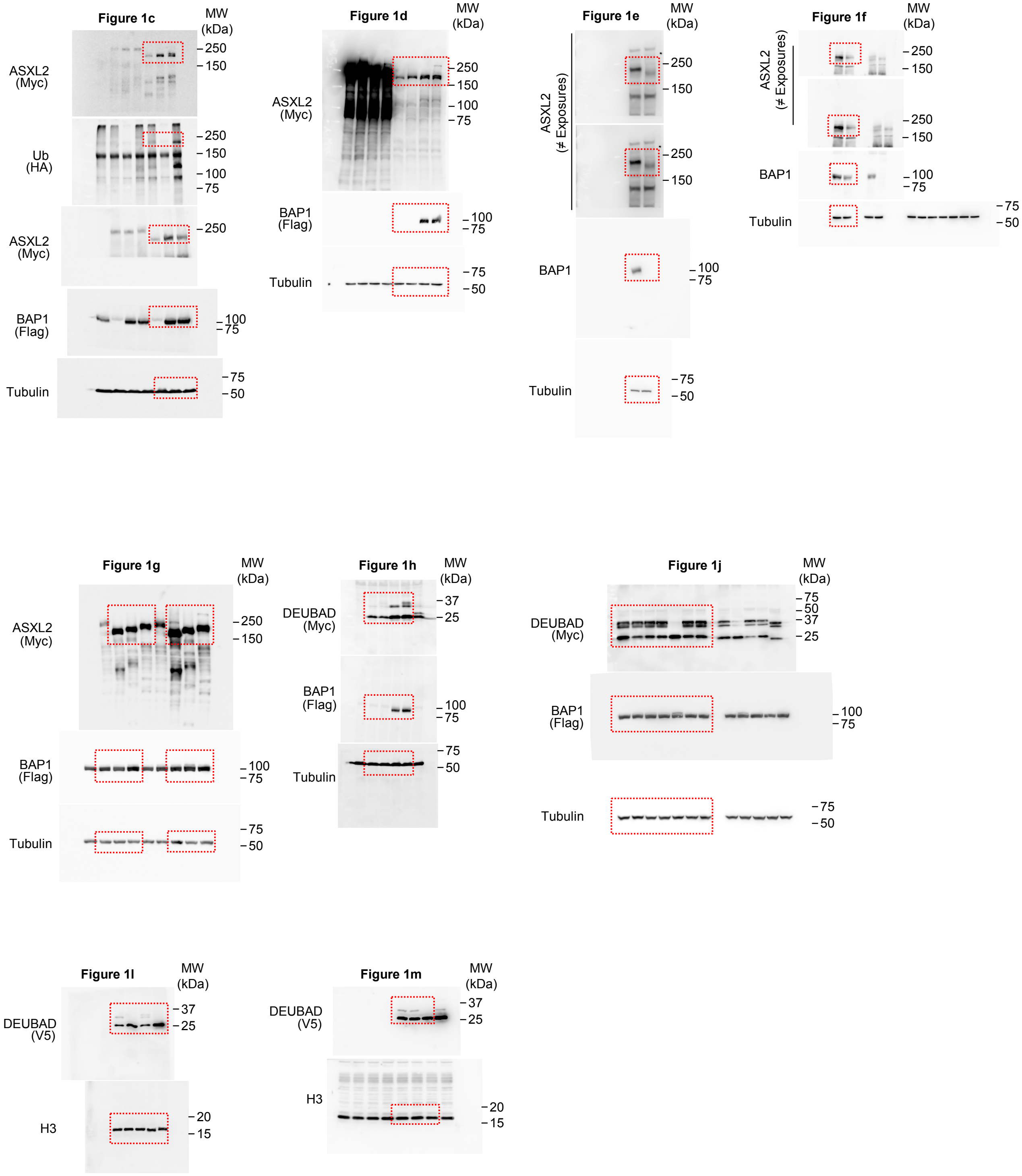

Supplementary Figure 13. Uncropped immunoblots used for the indicated proteins in the **Figure 2**

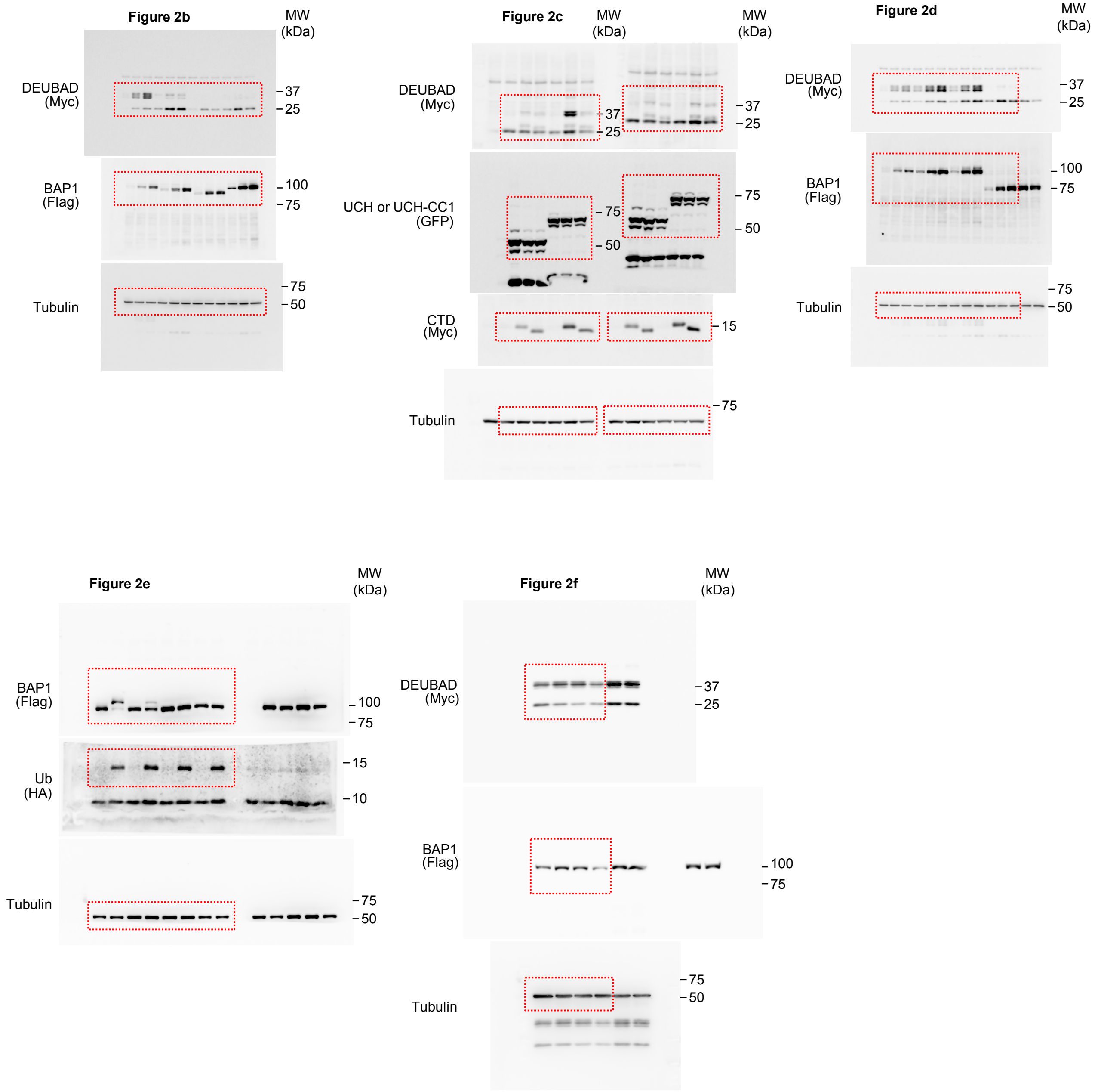

Supplementary Figure 14. Uncropped immunoblots used for the indicated proteins in the **Figure 3**

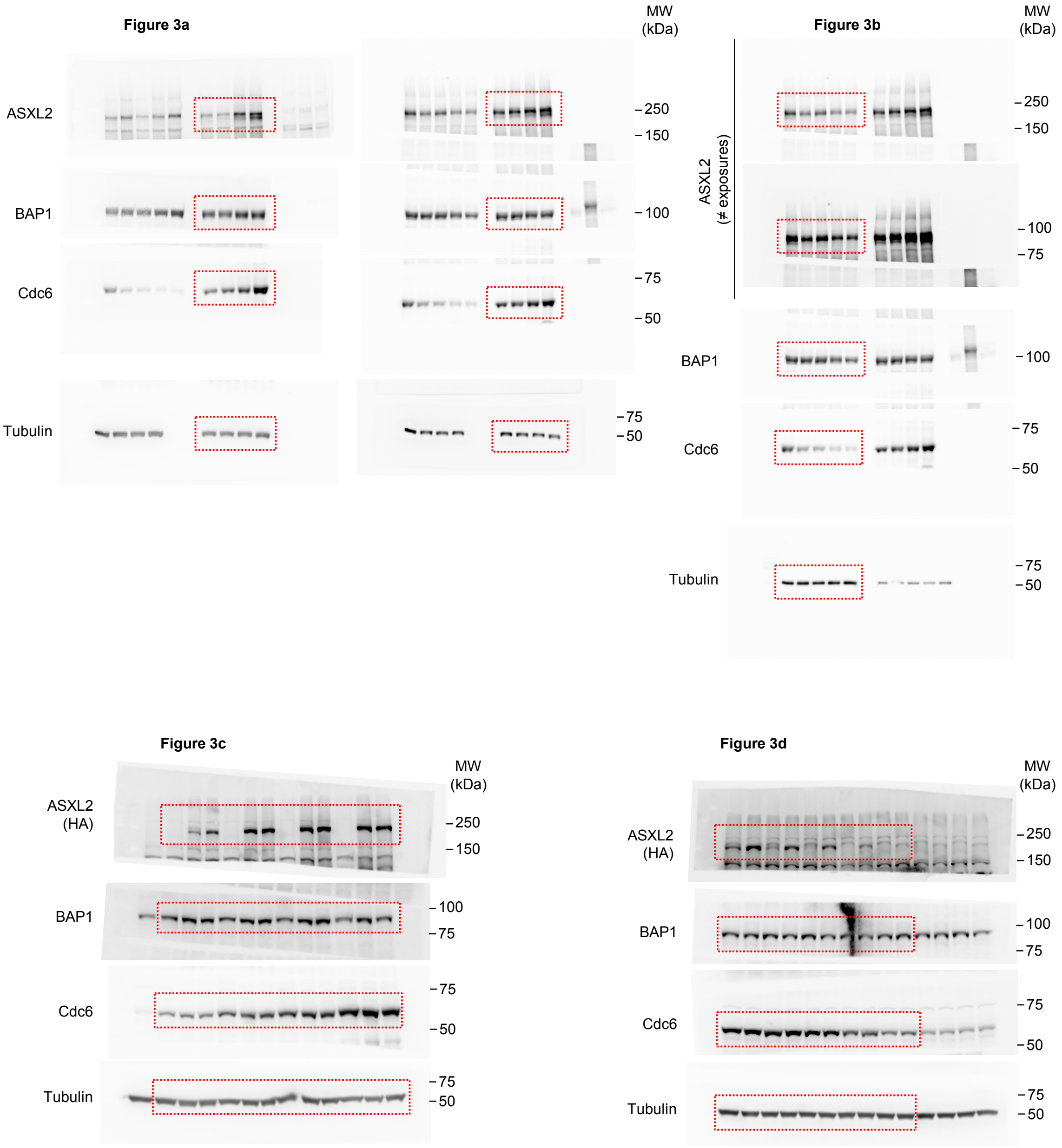

Supplementary Figure 15. Uncropped immunoblots used for the indicated proteins in the **Figure 3**

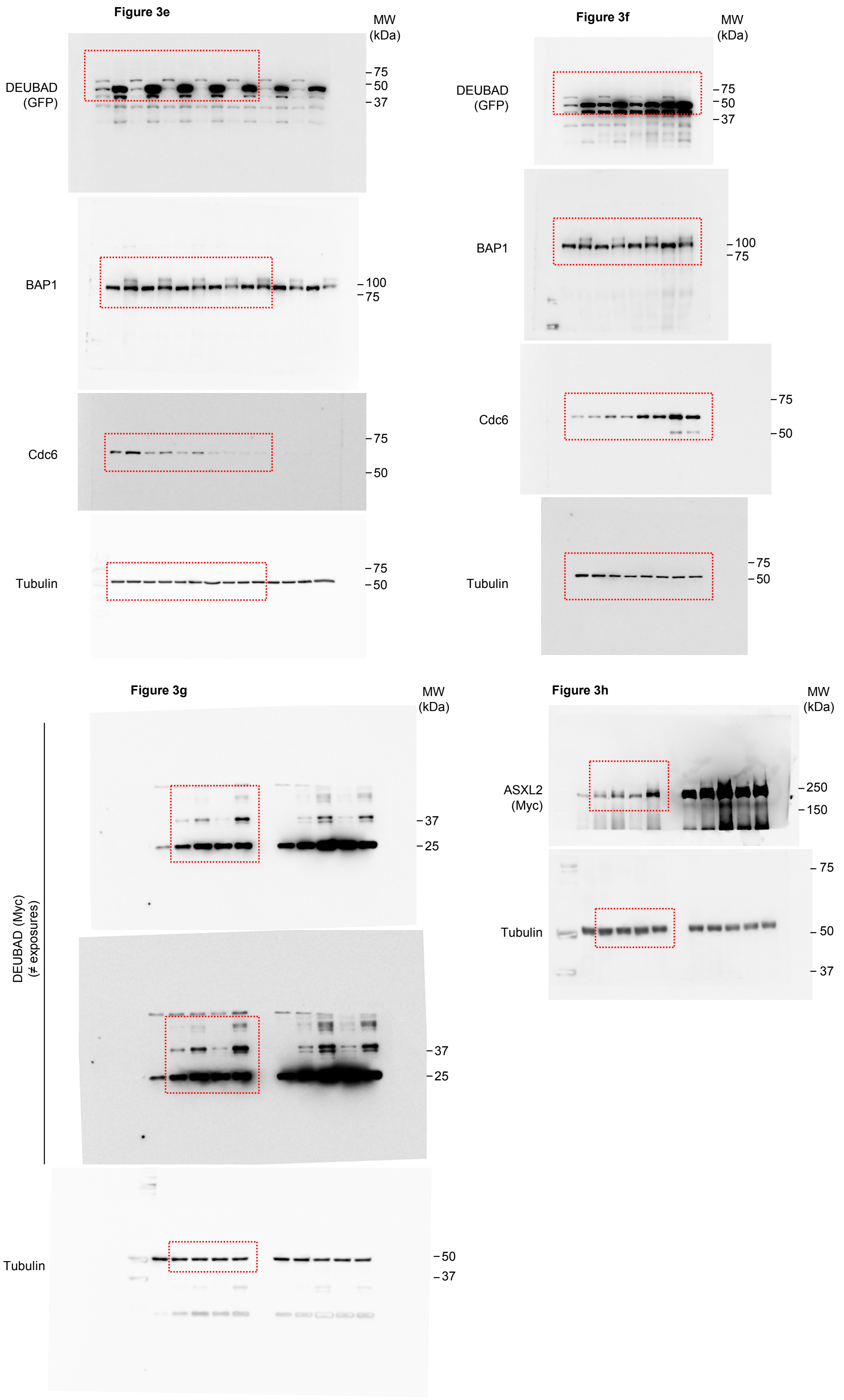

Supplementary Figure 16. Uncropped immunoblots used for the indicated proteins in the **Figure 4**

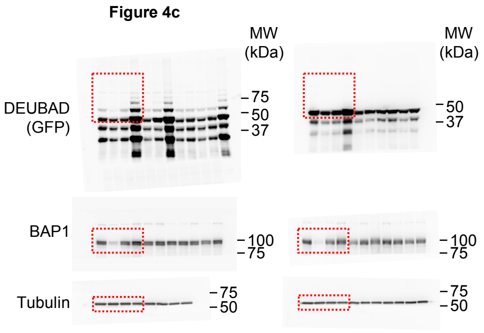

Supplementary Figure 17. Uncropped immunoblots used for the indicated proteins in the **Figure 5**

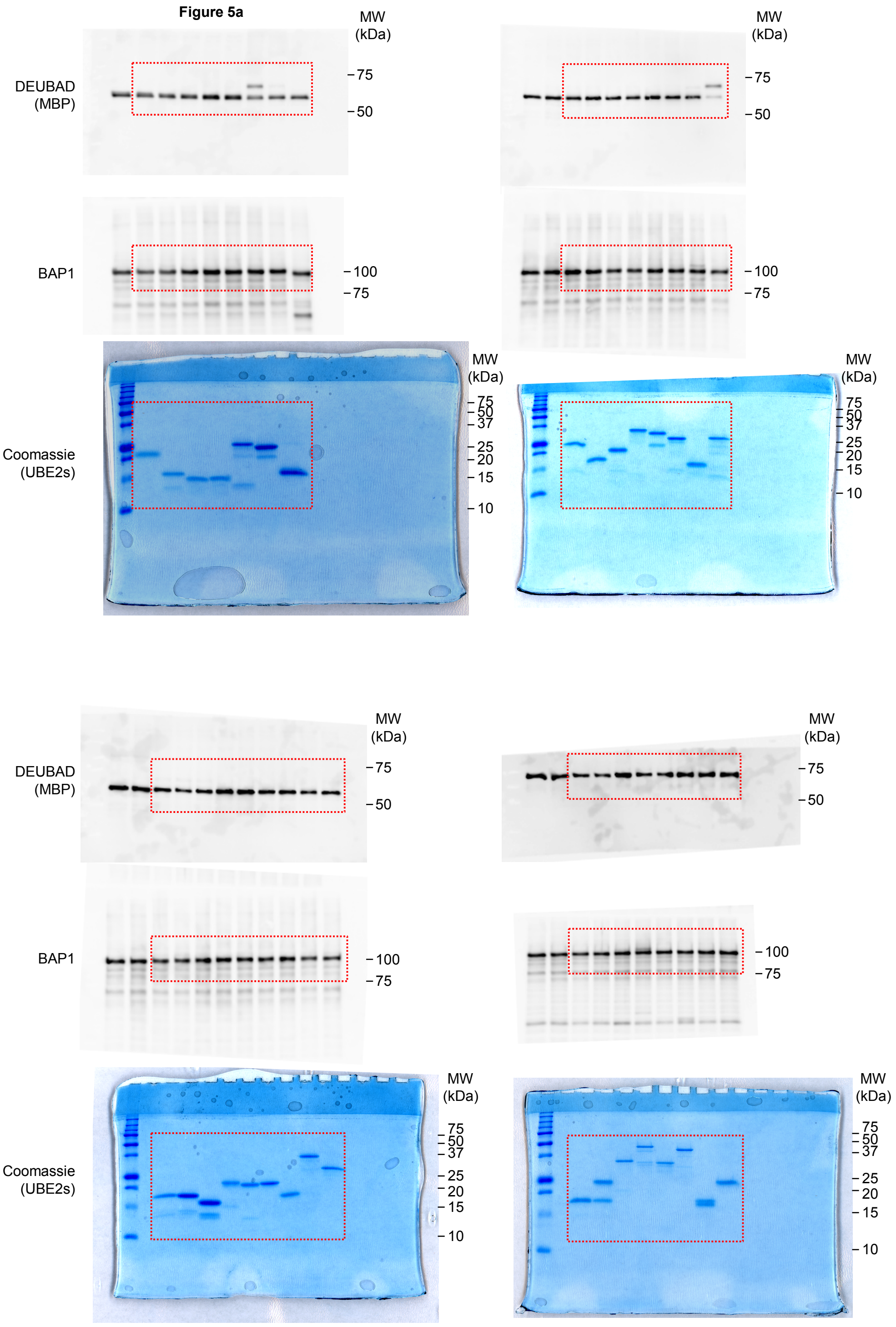

Supplementary Figure 18. Uncropped immunoblots used for the indicated proteins in the **Figure 5**

Figure 5b

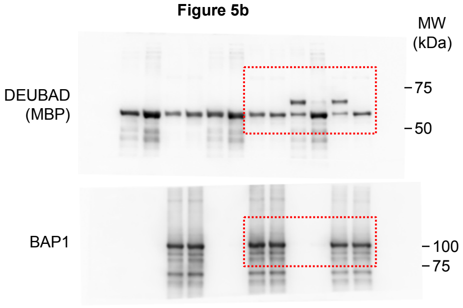

Figure 5d

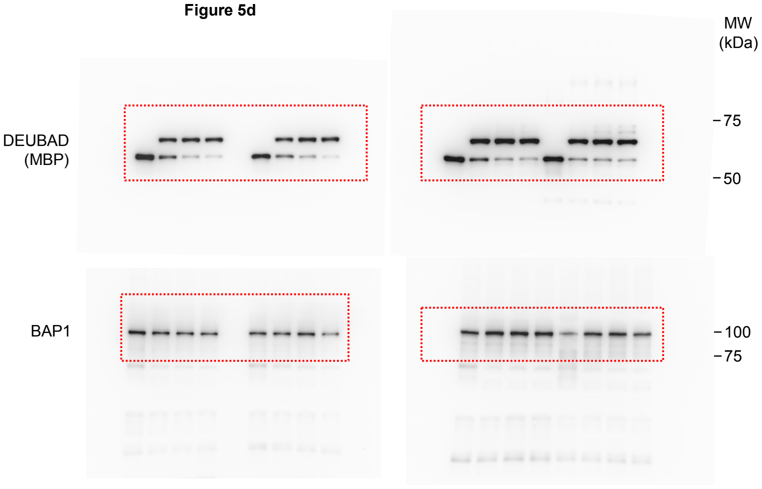

Figure 5c

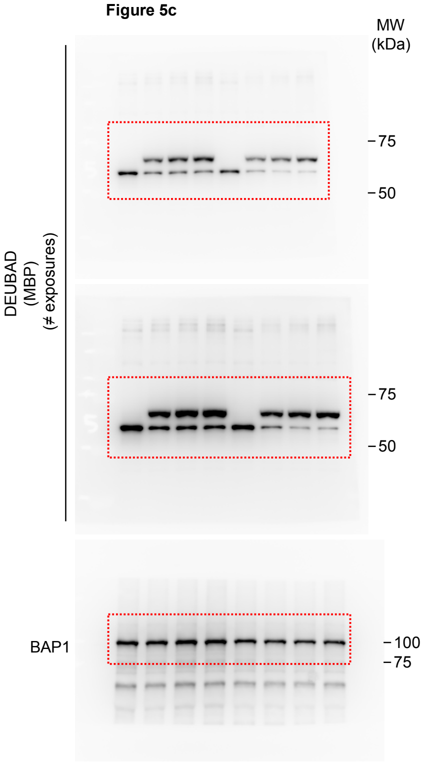

Supplementary Figure 19. Uncropped immunoblots used for the indicated proteins in the **Figure 6**

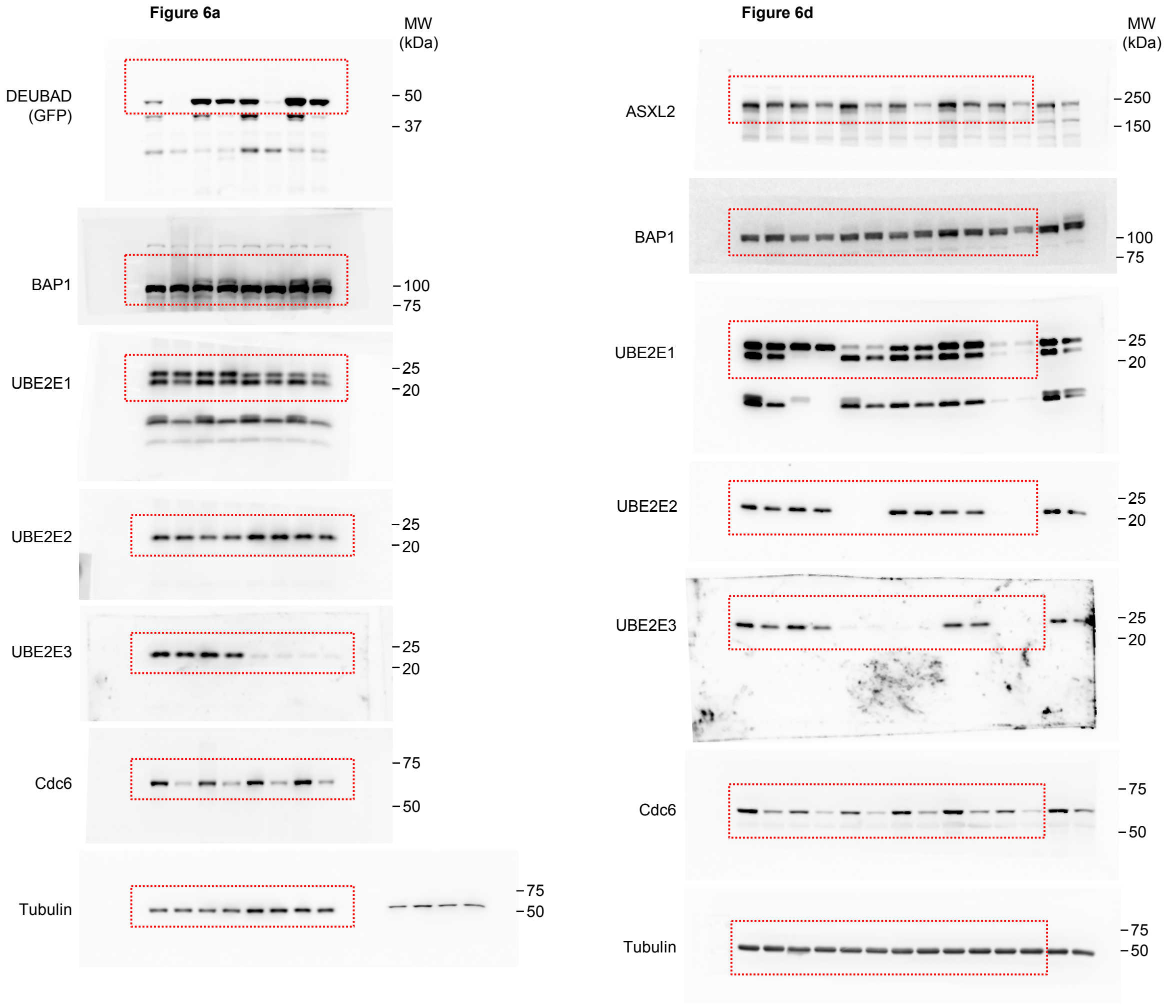

Supplementary Figure 20. Uncropped immunoblots used for the indicated proteins in the **Figure 6**

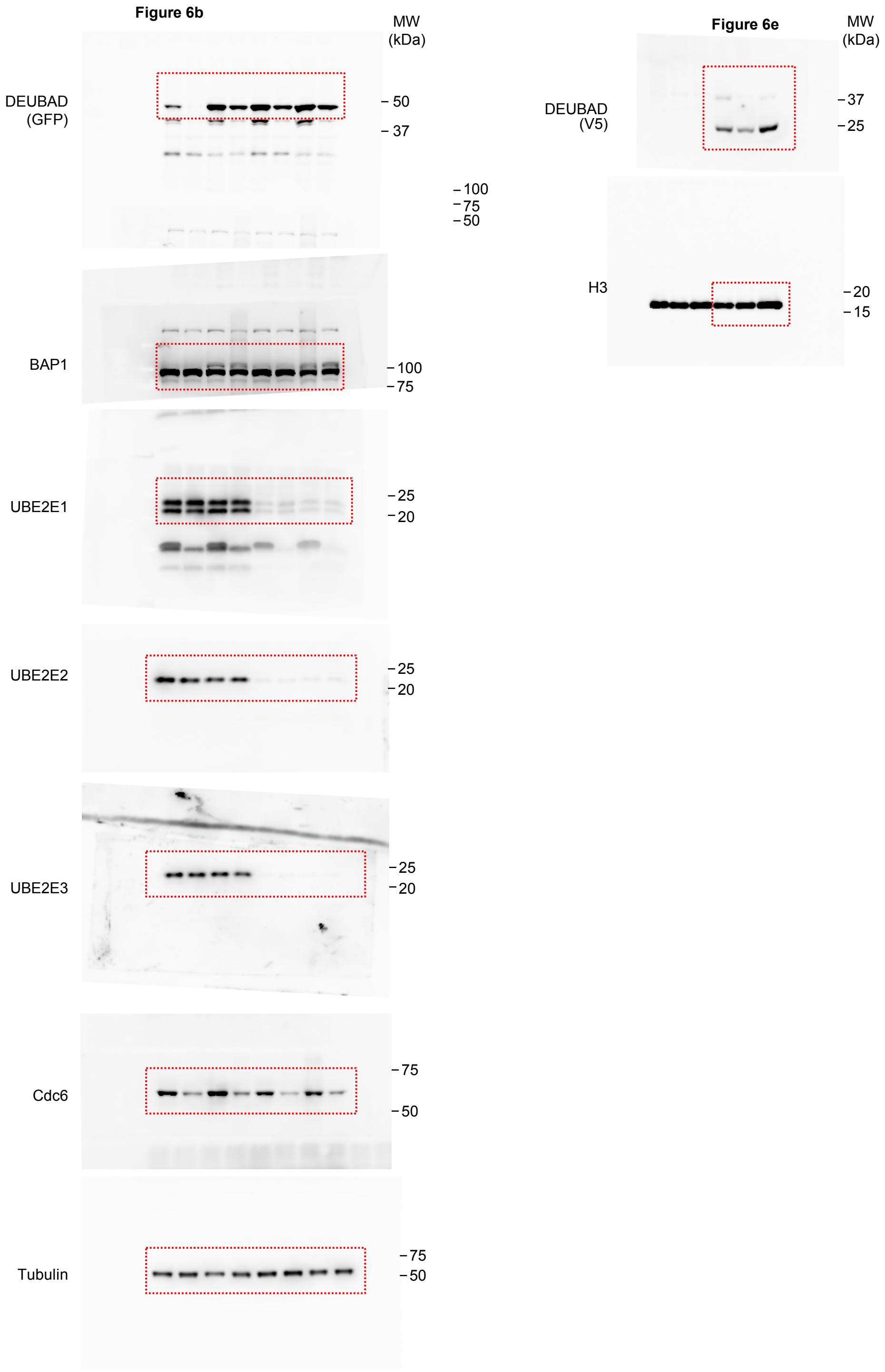

Supplementary Figure 21. Uncropped immunoblots used for the indicated proteins in the **Figure 6**

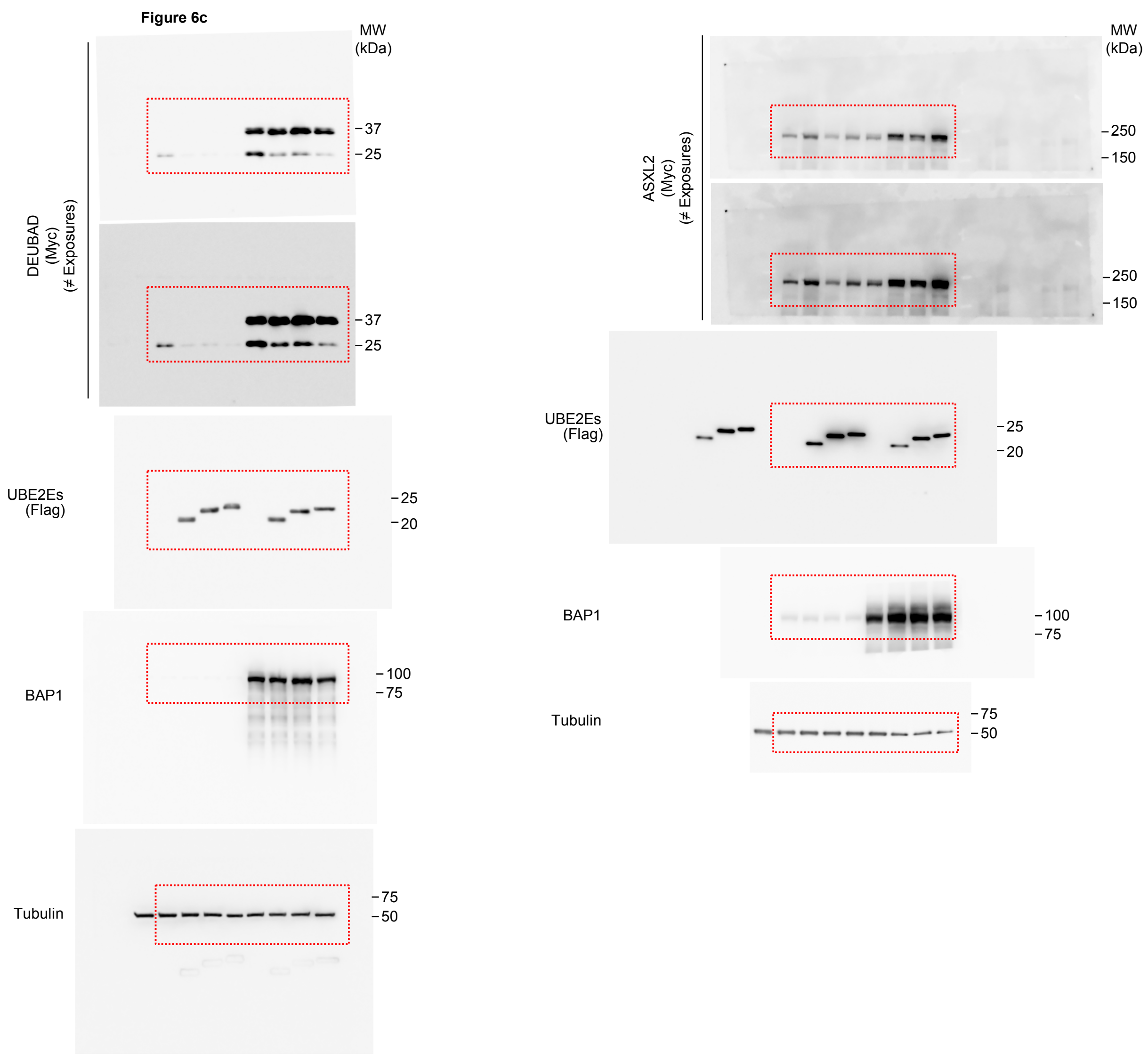

Supplementary Figure 22. Uncropped immunoblots used for the indicated proteins in the **Figure 7**

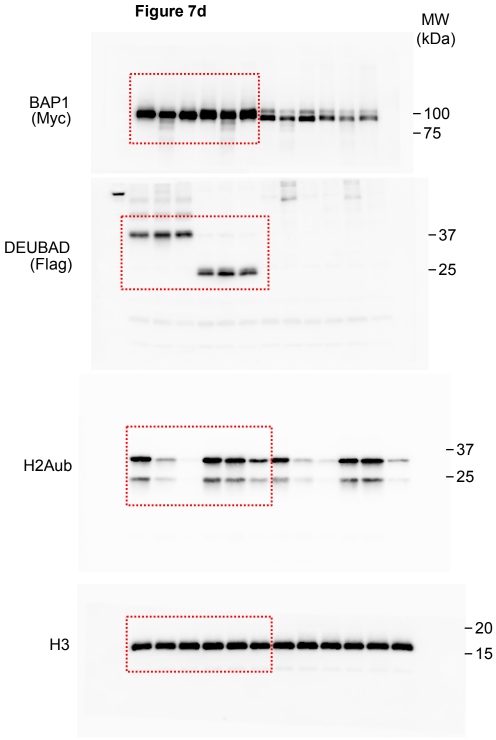

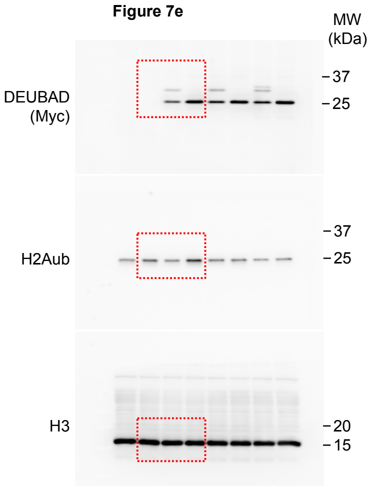

Supplementary Figure 23. Uncropped immunoblots used for the indicated proteins in the **Figure 9**

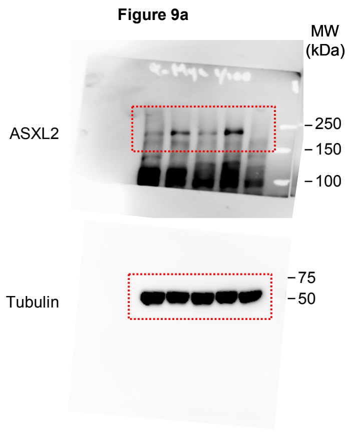

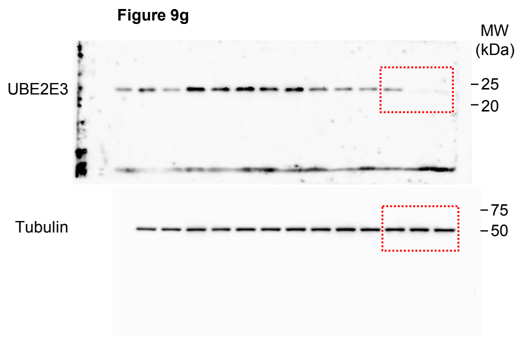

Supplementary Figure 24. Uncropped immunoblots used for the indicated proteins in the **Supplementary Figure 1**

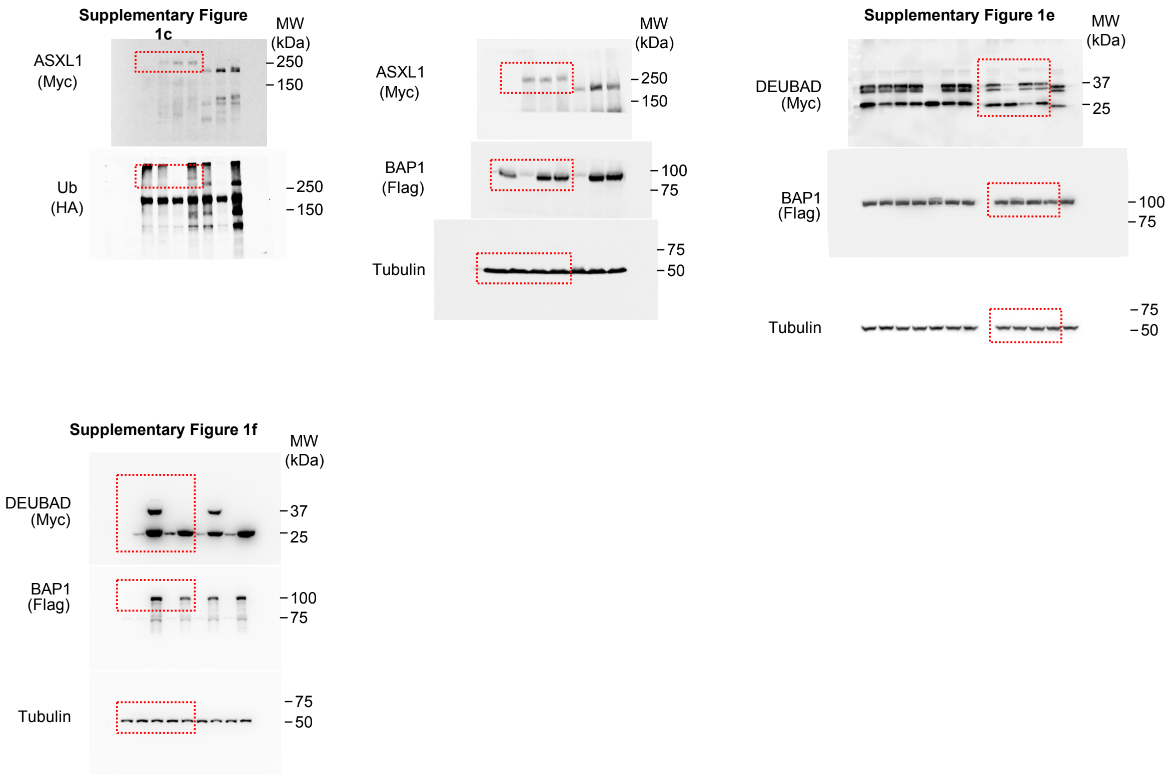

Supplementary Figure 25. Uncropped immunoblots used for the indicated proteins in the **Supplementary Figure 2**

Supplementary Figure 2a

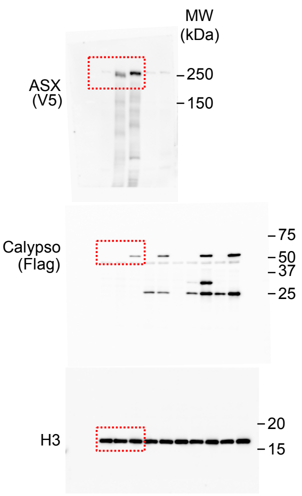

Supplementary Figure 2b

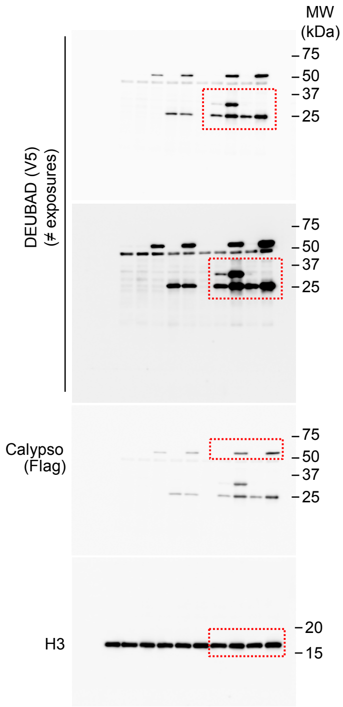

Supplementary Figure 2c

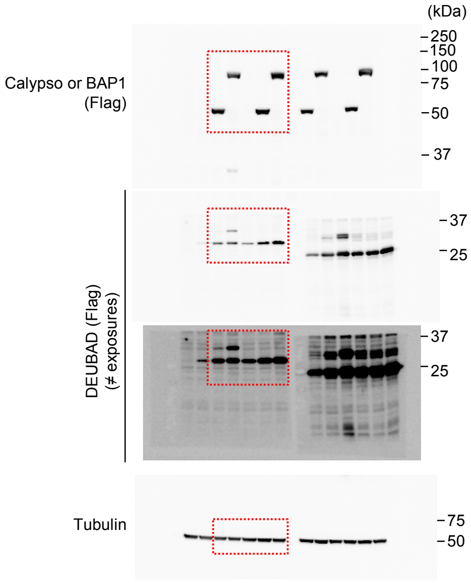

Supplementary Figure 2d

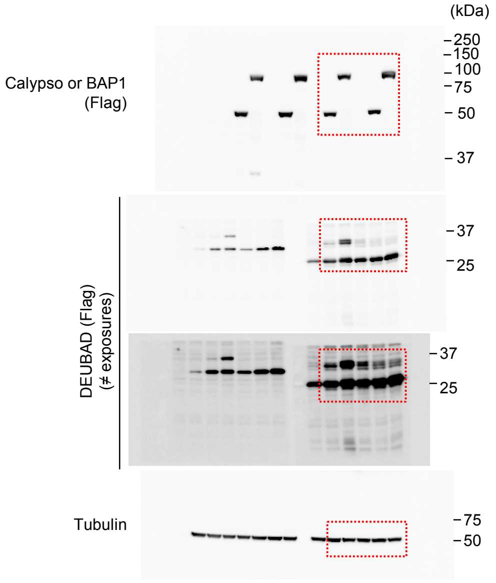

Supplementary Figure 26. Uncropped immunoblots used for the indicated proteins in the **Supplementary Figure 2**

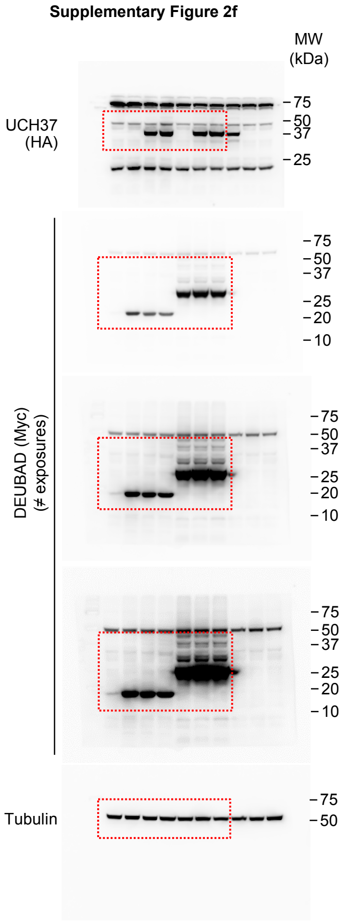

Supplementary Figure 27. Uncropped immunoblots used for the indicated proteins in the **Supplementary Figure 3**

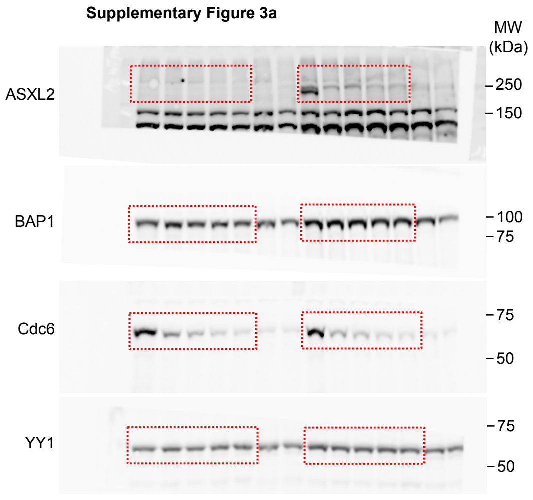

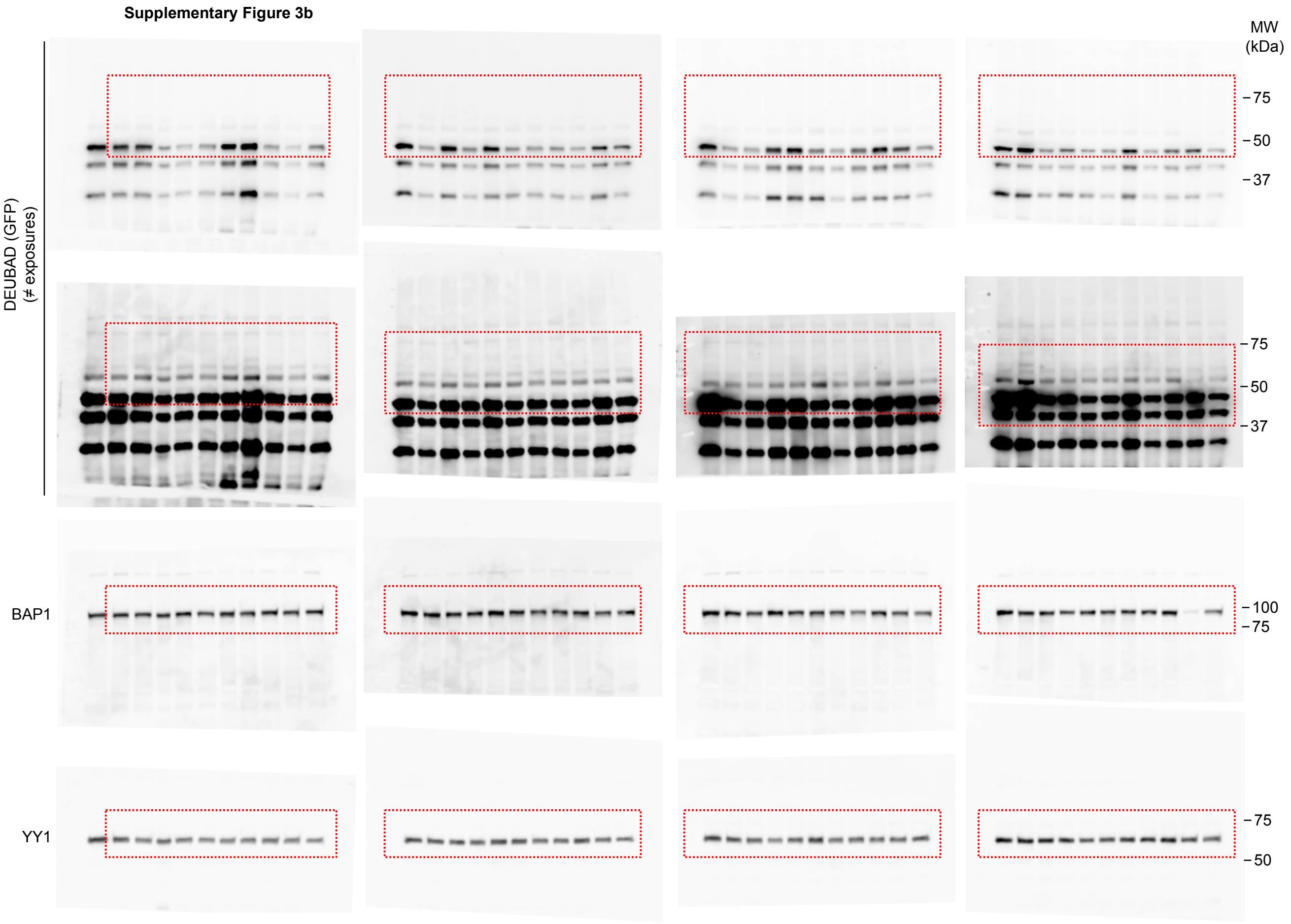

Supplementary Figure 28. Uncropped immunoblots used for the indicated proteins in the **Supplementary Figure 3b**

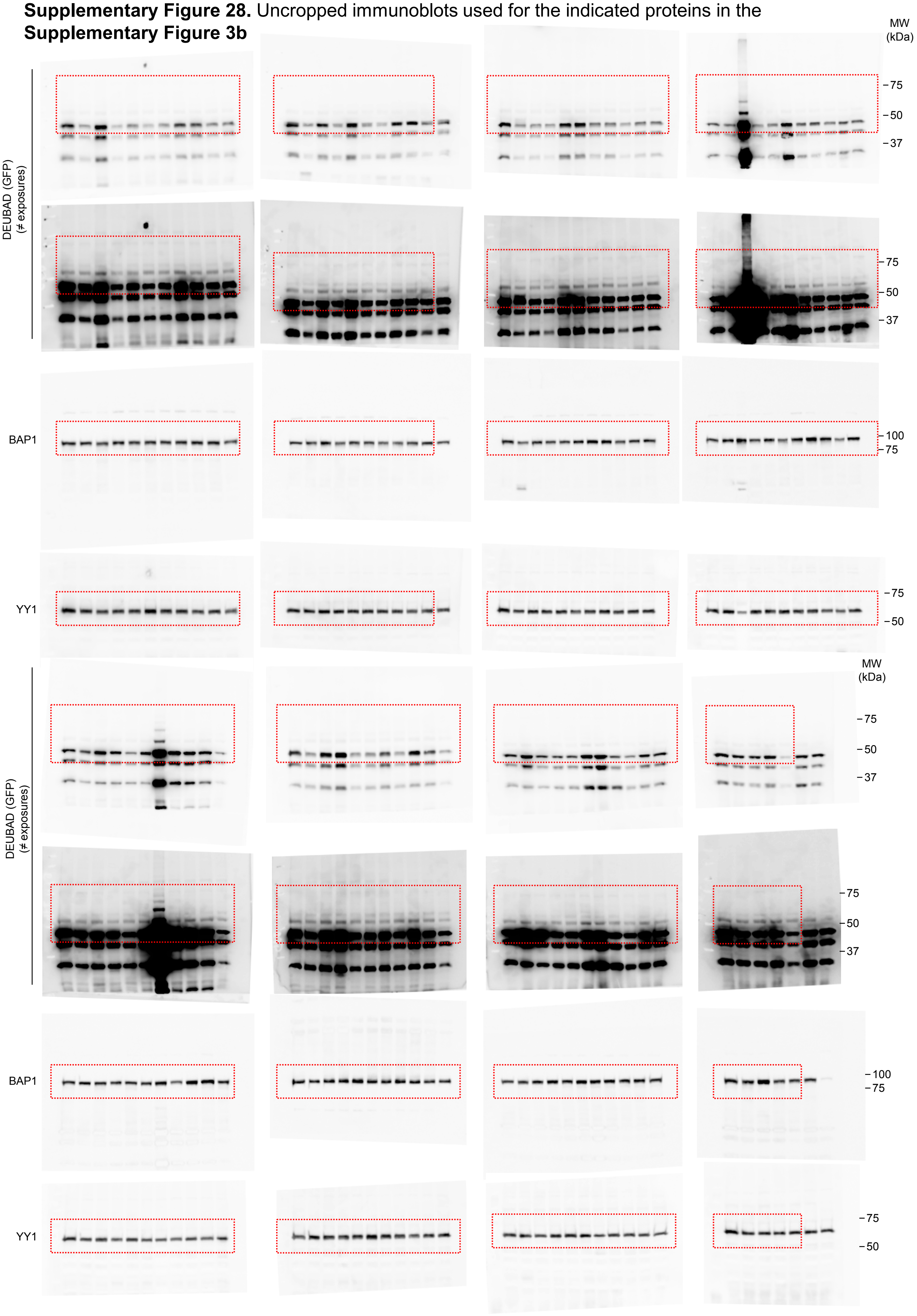

Supplementary Figure 29. Uncropped immunoblots used for the indicated proteins in the **Supplementary Figure 4**

Supplementary Figure 4b

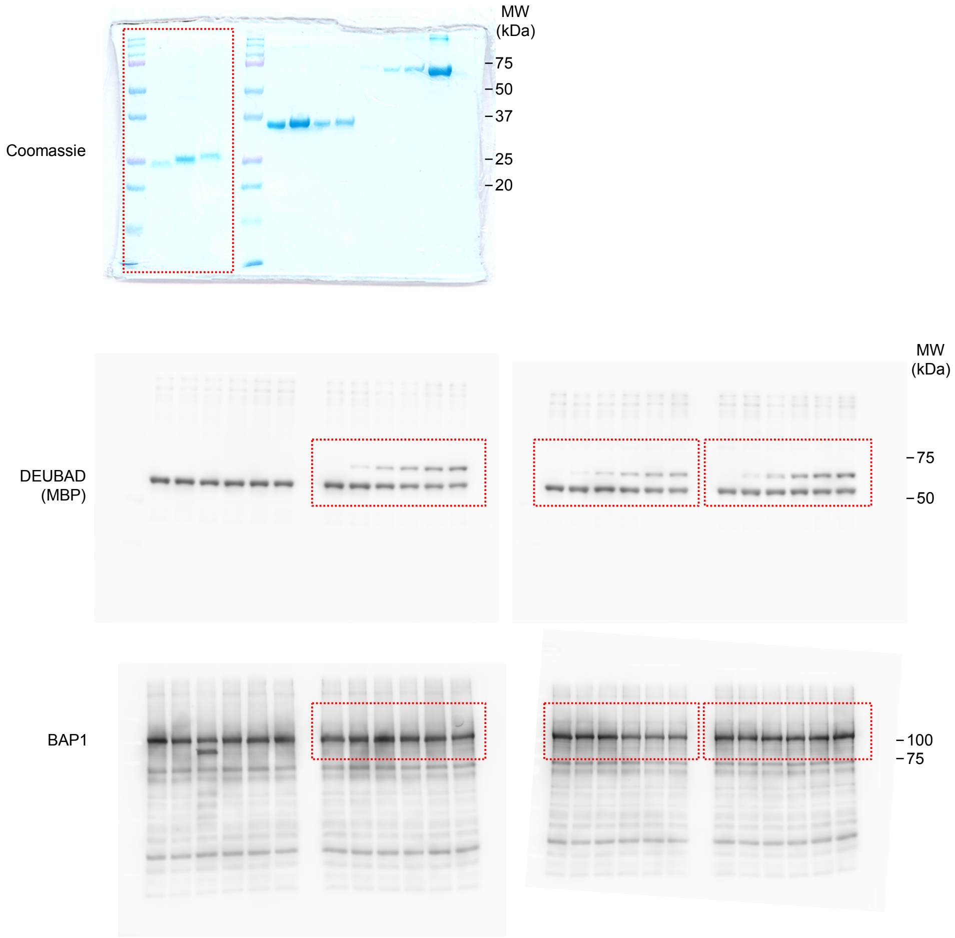

Supplementary Figure 30. Uncropped immunoblots used for the indicated proteins in the **Supplementary Figure 5**

Supplementary Figure 5a

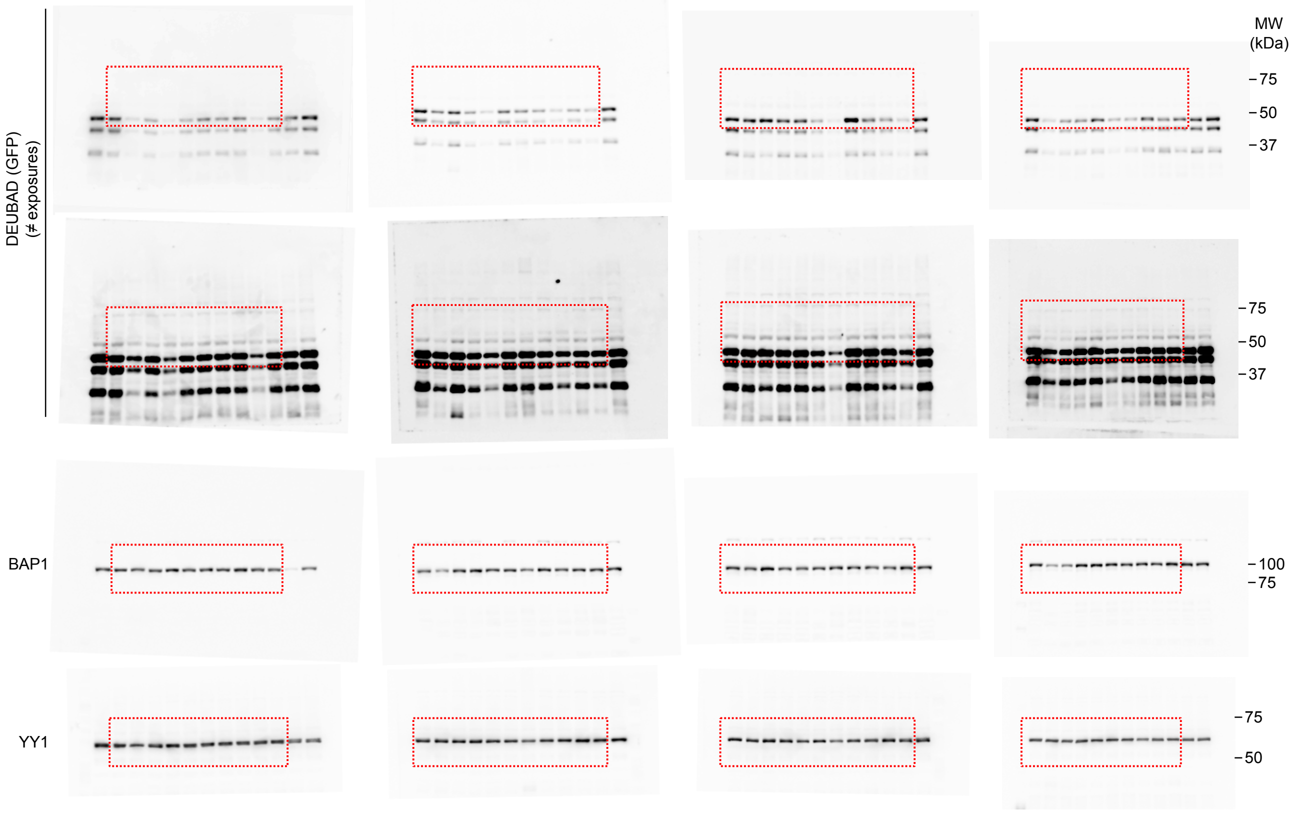

Supplementary Figure 5c

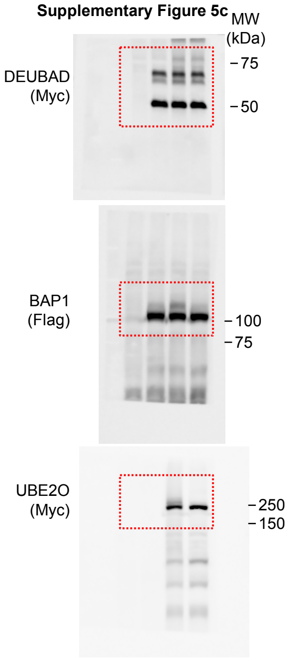

Supplementary Figure 5d

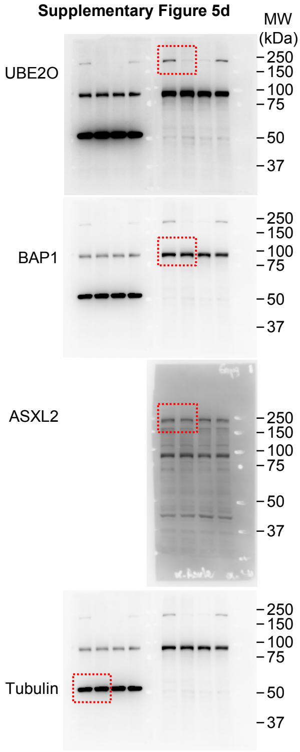

Supplementary Figure 5e

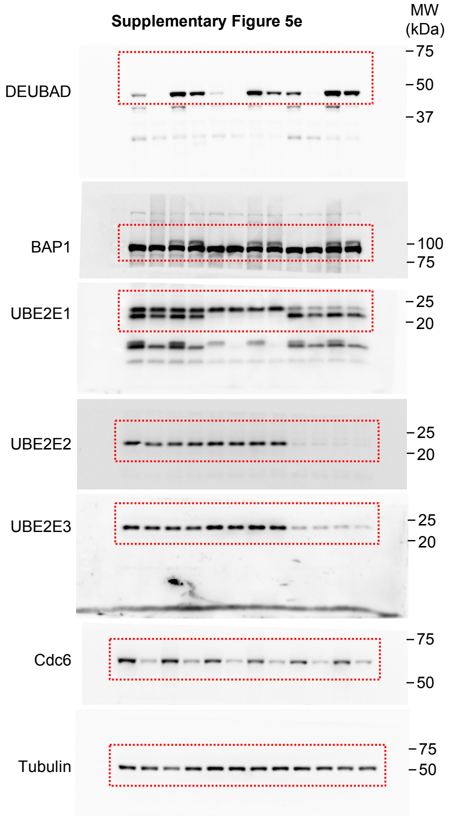

Supplementary Figure 31. Uncropped immunoblots used for the indicated proteins in the **Supplementary Figure 8**

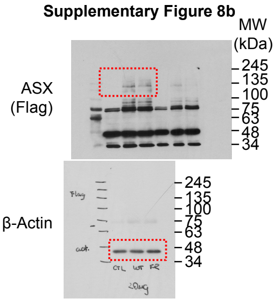

Supplementary Figure 32. Uncropped immunoblots used for the indicated proteins in the **Supplementary Figure 9**

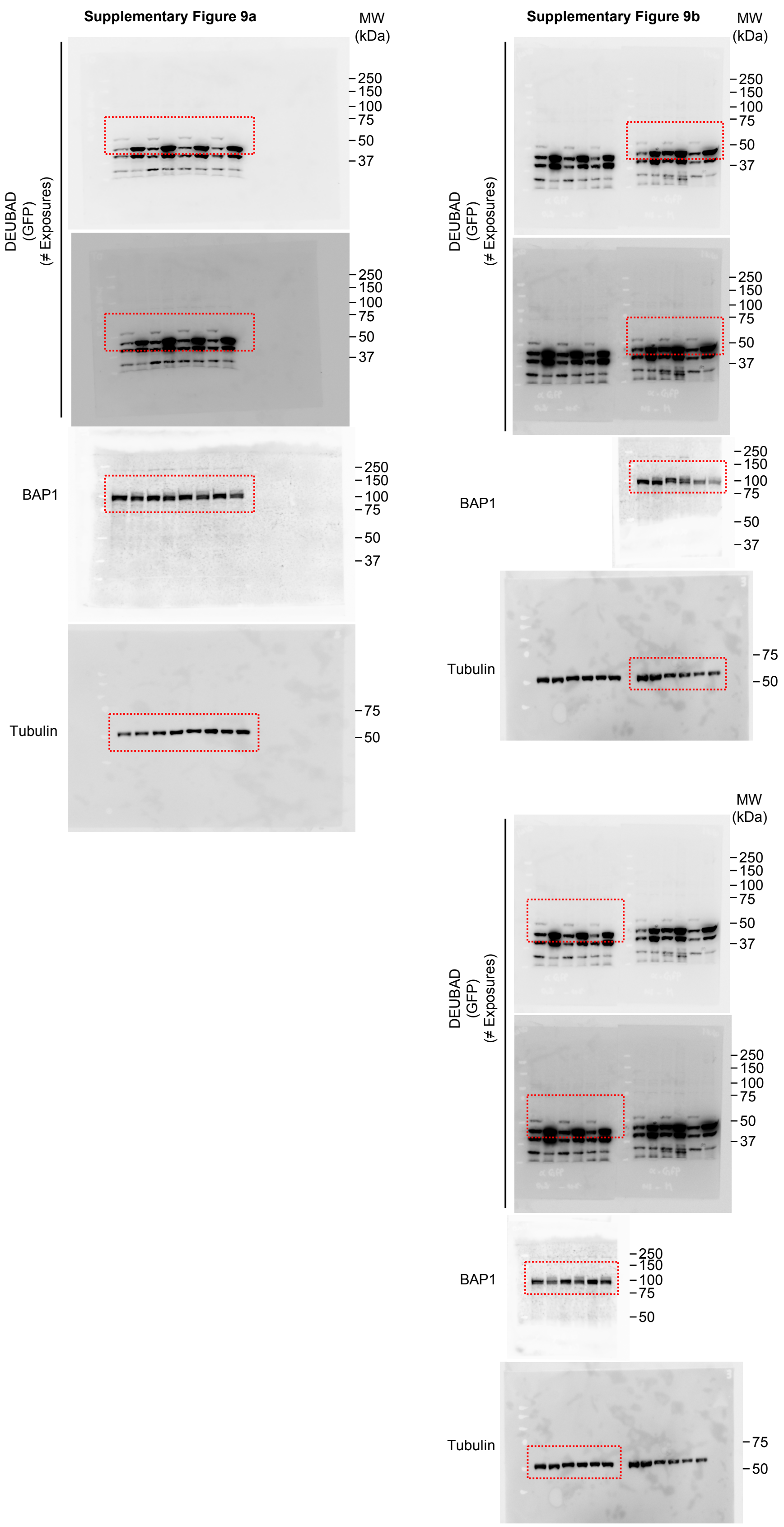

Supplementary Figure 33. Uncropped immunoblots used for the indicated proteins in the **Supplementary Figure 9**

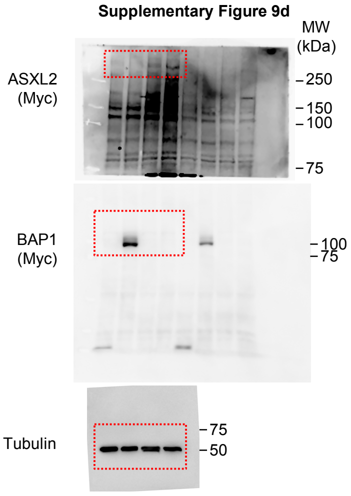

Supplementary Figure 34. Uncropped FACS plots used in the **Figure 9**

Figure 9b

MYC-GFP | Myc-ASXL2 | Myc-ASXL2 (+2X) | Myc-ASXL2 K370R (-2X) | Myc-ASXL2 K370R

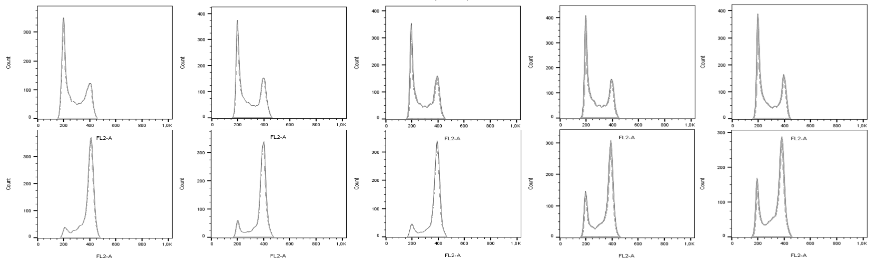

Figure 9e

siRNA Control | siRNA ASXL2 (1/2/3)

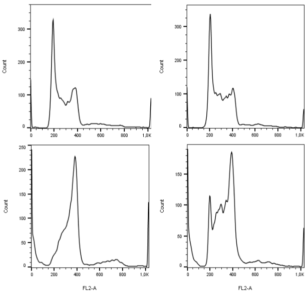

Figure 9i

gRNA Control | gRNA UBE2E3-1 | gRNA UBE2E3-2

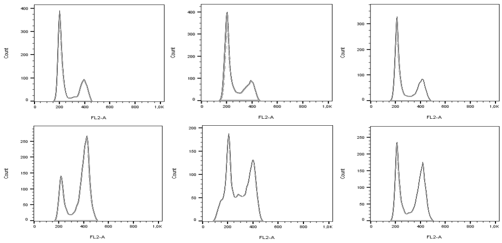

Supplementary Figure 35. Uncropped FACS plots used in the **Supplementary Figure 9**

Supplementary Figure 9a

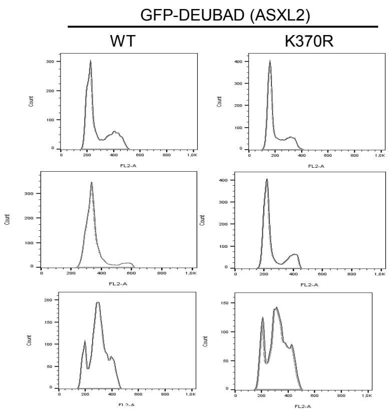

Supplementary Figure 9b

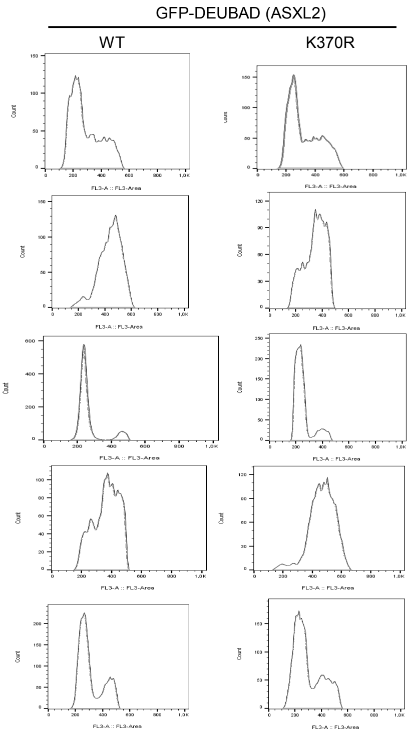

Supplementary Figure 36. Uncropped FACS plots used in the **Supplementary Figure 9**

Supplementary Figure 9f

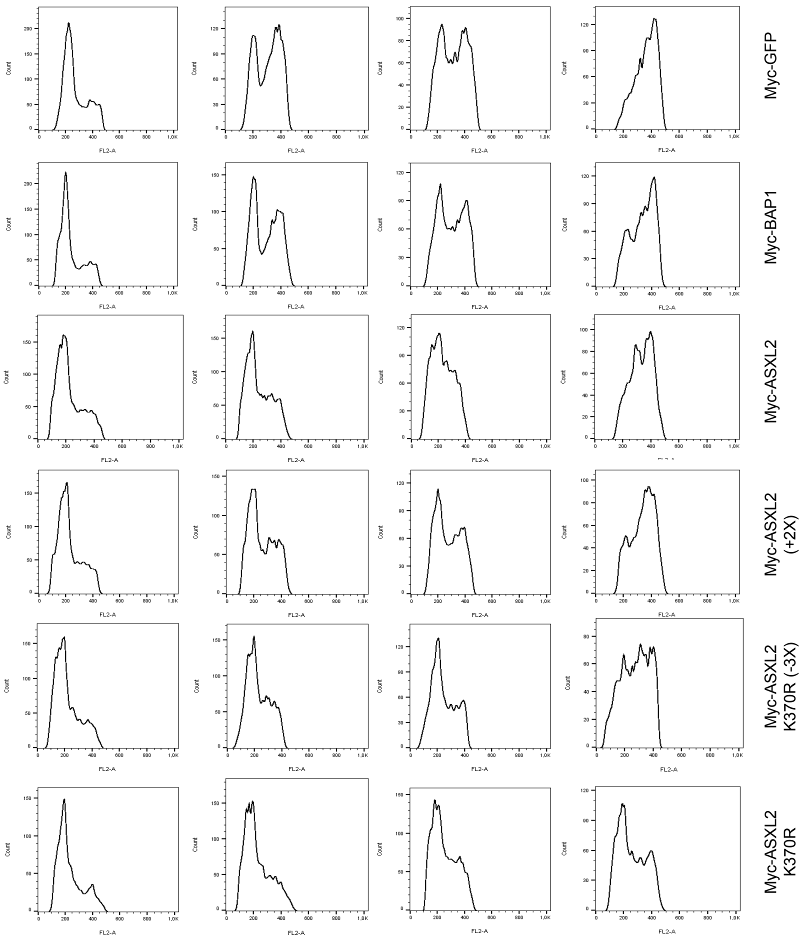

Supplementary Figure 37. Uncropped FACS plots used in the **Supplementary Figure 10**

Supplementary Figure 10b

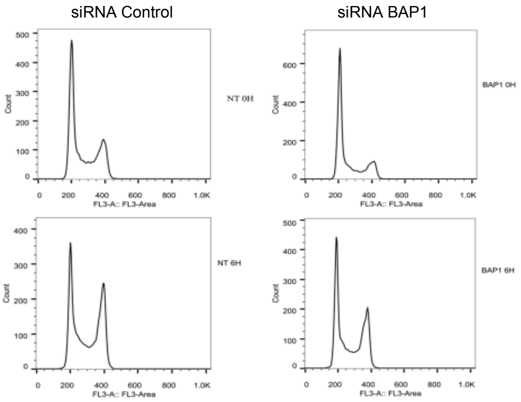

Supplementary Figure 10c

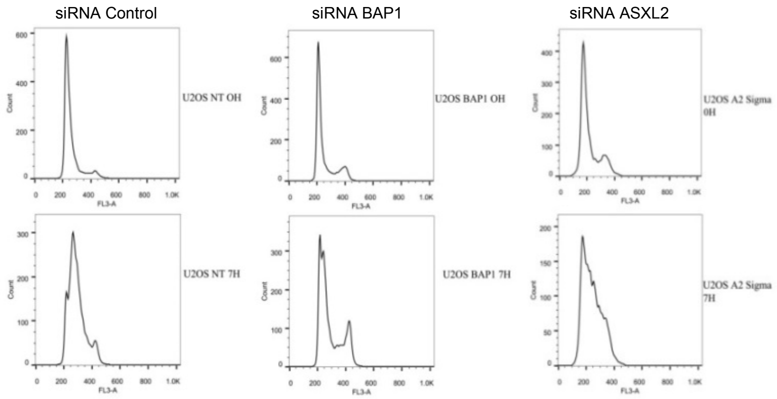

Supplementary Figure 38. Exemple of gating procedure related to **Figures 9b**, **9e**, **9i**, and **Supplementary Figures 9a**, **9b**, **9f**, **10b**, **10c**

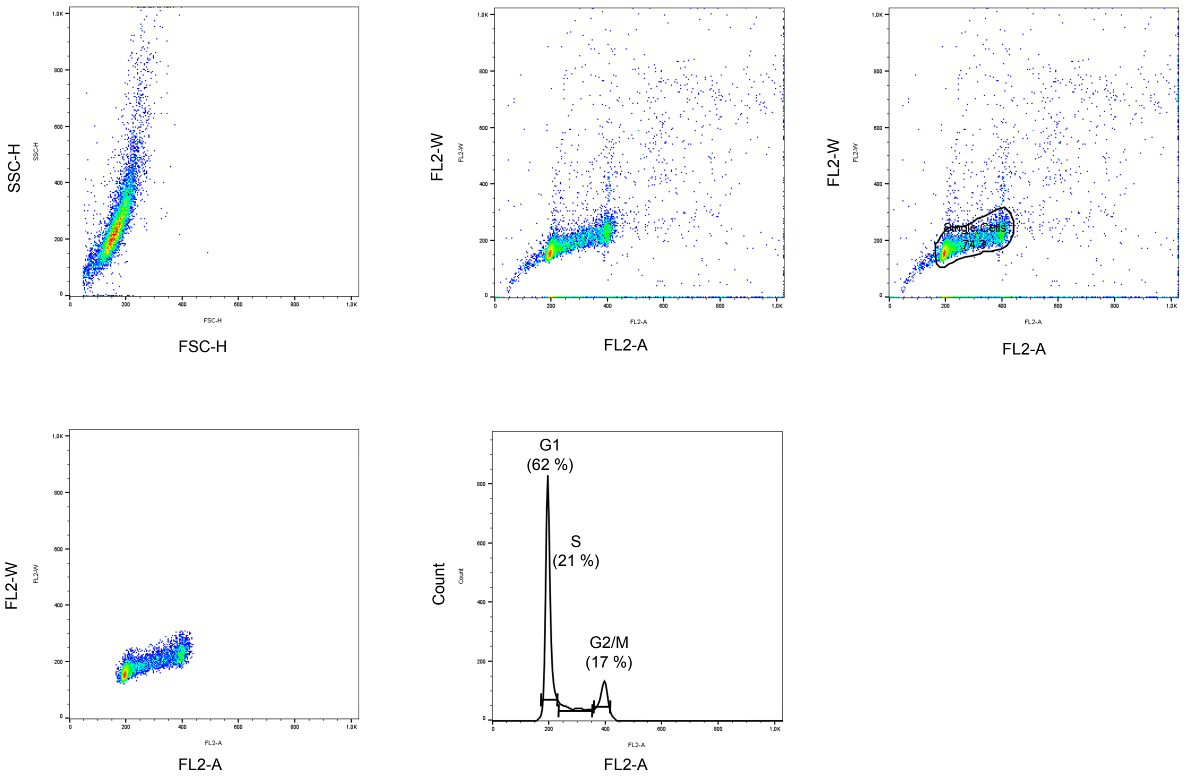

Supplementary Table 1 : DUB siRNA library

Target	siRNA_1	Name	siRNA_2	Name
**FAM63A**	CAGACUUGGUAAUGUCCUU	SASI_Hs01_00094199	GGGUGAACUUAGCGUCUUU	SASI_Hs01_00094200
**FAM63B**	CCAUCAUCACCCAGAAUGA	SASI_Hs02_00308987	CUCAGAAUUUCAUCUUCGA	SASI_Hs01_00060574
**FAM188A**	CACAGAUCGCUCUCCUUCA	SASI_Hs01_00110290	CCUUGAUAGAUCCUGUAUA	SASI_Hs01_00110291
**FAM188B**	CUCAUCACCUCAUCACCGA	SASI_Hs01_00139144	CUCUACUUGCCUGGUGGUA	SASI_Hs01_00139145
**BAP1**	CGUGAUUGAUGAUGAUAUU	SASI_Hs01_00105395	CCAUCAACGUCUUGGCUGA	SASI_Hs01_00105396
**UCHL1**	GGACAAGAAGUUAGUCCUA	SASI_Hs01_00178415	GGCCAAUAAUCAAGACAAA	SASI_Hs01_00178416
**UCHL3**	CUGAUUCAUGCUAUUGCAA	SASI_Hs01_00200423	GCAUCUCUAUGAAUUAGAU	SASI_Hs01_00200424
**UCHL5**	CAGUUAUGUUCCUGUUAAU	SASI_Hs01_00142742	GAAGCAUAAUUAUCUGCCU	SASI_Hs01_00142743
**USP1**	GUAUACUUCAGGUAUUAUA	SASI_Hs01_00204271	CCAUACAAACAUUGGUAAA	SASI_Hs01_00204272
**USP2**	GACCUAAGUCCAACCCUGA	SASI_Hs01_00149958	CUAAGAGACCUGGACUUAA	SASI_Hs02_00337104
**USP3**	CGGAUAAACUUUAAUACCU	SASI_Hs01_00023593	CAUUACACAGCAUACGCAA	SASI_Hs01_00023595
**USP4**	CACUACACUGCAUAUGCGA	SASI_Hs01_00113975	GAGAAUCACAGGUUGAGGA	SASI_Hs01_00113976
**USP5**	CUGUCAAGCUGGGCACCAU	SASI_Hs02_00319396	CUAAUGAAGUGUUCCGCUU	SASI_Hs02_00319397
**USP6**	GUGUUGAUGCCAAUAACCA	SASI_Hs01_00167628	GAGAAUGGGAGACAUAUAA	SASI_Hs01_00167629
**USP7**	GACGUUUCGAAUAGAGGAA	SASI_Hs01_00079539	GACUUUGAGAACAGGCGAA	SASI_Hs01_00079540
**USP8**	CCUUUGACAAGAGCACGAA	SASI_Hs02_00339089	GAGAAUGGGACCACUGAAU	SASI_Hs01_00136039
**USP9X**	GUCGUUACAGCUAGUAUUU	SASI_Hs01_00026227	CUGUGAUUCAGCAACUCUA	SASI_Hs02_00308595
**USP9Y**	GUAGUGAUUUACACGAUGA	SASI_Hs01_00081909	CUUACUAAGAGCCACACUA	SASI_Hs02_00338066
**USP10**	GUCAUUGAACCCAGUGACA	SASI_Hs01_00213007	GUUCUAAUGUGGAGGCGGA	SASI_Hs01_00213008
**USP11**	CAGAGAUGAAGAAGCGUUA	SASI_Hs01_00148685	GAUUCUAUUGGCCUAGUAU	SASI_Hs01_00148686
**USP12**	GAAACUCUGUGCAGUGAAU	SASI_Hs01_00167303	CAUCAGAUAUCUCAAAGAA	SASI_Hs01_00167305
**USP13**	CUGAAUACUUGGUAGUGCA	SASI_Hs01_00108438	GAGCUAUUUGCAUUCAUCA	SASI_Hs01_00108439
**USP14**	CAAUAAUUGUGGAUACUAU	SASI_Hs01_00089059	GAUAUUGGCUCCAAUAAUU	SASI_Hs01_00089060
**USP15**	CUCUUGAGAAUGUGCCGAU	SASI_Hs01_00059894	CACAAUAGAUACAAUUGAA	SASI_Hs01_00059895
**USP16**	CAUCUUUGGUGGUGAACUA	SASI_Hs01_00210886	GAGAAACUUCGAGAUGCGA	SASI_Hs02_00304059
**USP17L9P**	GAAAUUCCUUCAAGAGCAA	SASI_Hs02_00324631	GGAAAUUCCUUCAAGAGCA	SASI_Hs02_00324632
**USP17L2**	CUAUCAUUGCGGUCUUUGU	SASI_Hs02_00372940	CAACAAACUUGCCAAGAAU	SASI_Hs02_00372941
**USP17L6P**	GCCUAUCAUUGUGGUGUUU	SASI_Hs02_00494866	CAGGCAACAAGAUUGCCAA	SASI_Hs02_00494867
**USP17L1P**	CUAUCAUUGCGGUCUUUGU	SASI_Hs02_00517496	GCAACAAACUUGCCAAGAA	SASI_Hs02_00517497
**USP18**	GCUUCAAUGACUCCAAUAU	SASI_Hs01_00221412	GUCAUUACUGUGUCUACAU	SASI_Hs01_00221413
**USP19**	CACAAGAUGAGGAAUGACU	SASI_Hs01_00130241	GCAAGUUCUGCAUUGGUCA	SASI_Hs01_00130242
**USP20**	GGACUUAUCACUGCCCAUU	SASI_Hs01_00033438	GGUUCUACGUGUCCCGCGA	SASI_Hs01_00033439
**USP21-Iso-1**	CCAACUUAGCCCGUUCCAA	SASI_Hs01_00193567	GACAAGAUGGCUCAUCACA	SASI_Hs01_00193568
**USP21-Iso-2**	CCAACUUAGCCCGUUCCAA	SASI_Hs01_00177787	GACAAGAUGGCUCAUCACA	SASI_Hs01_00177788
**USP22**	CAAAGCAGCUCACUAUGAA	SASI_Hs02_00347438	CUGAUCAACCUUGGGAACA	SASI_Hs02_00347439
**USP24**	GCACAAUACUGUGACCGUA	SASI_Hs02_00347524	GAAACUCAGGGUUGAUACU	SASI_Hs02_00347525
**USP25**	CUAUGGUUCCGGUCCCAAA	SASI_Hs02_00344762	GAAAGAUUACCUCACGGUA	SASI_Hs01_00191397
**USP26**	CUACAGAAGUCUAACAGGA	SASI_Hs01_00043149	CCAUCUUGGGAAGACUCUA	SASI_Hs01_00043150
**USP27X**	GAUACUGAGAGAUUUCUUU	SASI_Hs02_00394607	CCUGUAUUACGGAGGUAUA	SASI_Hs02_00394608

**USP28**	GACCUUACUCAUGAUAACA	SASI_Hs01_00077918	GACACUAUUGGGCCUAUAU	SASI_Hs01_00077919
**USP29**	GAAAGAAGCUCUCAUUGAA	SASI_Hs01_00032719	GAAUAACGAGCAAGUUUAU	SASI_Hs01_00032720
**USP30**	CACGAAUUAUUCCAUGUCA	SASI_Hs01_00155679	CUAGUCAACACAACCCUAA	SASI_Hs01_00155680
**USP31**	GCAUUCAGGUGUGUCCAUU	SASI_Hs01_00020641	GAGUCAUCCCUUUCAAGUA	SASI_Hs01_00020642
**USP32**	GAUAAUCAGCCAUUAGUAA	SASI_Hs01_00086230	GGAACUAUGUUAUACGGGA	SASI_Hs01_00086231
**USP33**	CACAGAUCCUUCCAUCAAA	SASI_Hs01_00180288	GAAGUGUUAUUUCAGACAU	SASI_Hs01_00180289
**USP34**	GGAUCUAGCAAUGAGGUUA	SASI_Hs01_00089169	GAUCUUAGGGCUGAAGUAA	SASI_Hs02_00346129
**USP35**	GCAAGAUUGGUCUCAUCAA	SASI_Hs02_00353962	CUGUUAAGAAGUUCAGCAU	SASI_Hs02_00353963
**USP36**	CUAAGACGGUGAAGCUGAA	SASI_Hs02_00357787	CGUAUAUGUCCCAGAAUAA	SASI_Hs01_00194136
**USP37**	CAGCUAAGUCAUAACAUUA	SASI_Hs01_00018875	CUUGUCUAUUGACAAAGUA	SASI_Hs02_00354323
**USP38**	GAGAGAUAGUCCCAGUGCA	SASI_Hs01_00189218	GUGAAACUUCUUUACAGGA	SASI_Hs02_00360065
**USP39**	CAUAUGAUGGUACCACUUA	SASI_Hs01_00034674	CAAUGAUUAUGCCAACGCU	SASI_Hs01_00034675
**USP40**	CACUGAAAGAACUUCUGAU	SASI_Hs01_00145783	CACAUGUCUUUCCAGCUAA	SASI_Hs02_00351527
**USP41**	GUACGUGCAUCCUUGUGUA	custom made by Sigma	UUGUUCAGGGCUCAUCAGU	custom made by Sigma
**USP42**	CAGUCUACCUCGAACGCAU	SASI_Hs01_00078970	GUUAAUAGGUCCUCAGUGA	SASI_Hs01_00078971
**USP43**	GUGAUCUUGGUUGAACUGU	SASI_Hs02_00367061	GUGAAAGGCAGAAGCAUUA	SASI_Hs02_00367062
**USP44**	GAAGGAUACUAAUGGGUAA	SASI_Hs02_00309548	CAACAAAUCAAAUACCAUA	SASI_Hs02_00309549
**USP45**	CCAGUUUACAUCUAUGGAA	SASI_Hs02_00315256	CACUACACUGCUUAUGUGA	SASI_Hs02_00315257
**USP46**	GUCUCAAUGGUCUGGCUGU	SASI_Hs01_00080807	GGUAUCACUCCGAGUCUCA	SASI_Hs01_00080808
**USP47**	GCAUAUAUGCUGAUCUAUA	SASI_Hs01_00112225	CAUGCAAGUUUCUGCUAGA	SASI_Hs01_00112227
**USP48**	CUUUAUGUCUCUAUUGGAA	SASI_Hs01_00185218	CUACUUAUGUCCAAGCACU	SASI_Hs01_00185219
**USP49**	CUGAAACACUUUGAGGAGA	SASI_Hs01_00055585	CUCAGAAGUGGUGCUGCUU	SASI_Hs02_00352112
**USP50**	CACUACACUGCUUUCUGCA	SASI_Hs02_00373416	GGAAAUAUCCUAAAUACAA	SASI_Hs02_00373417
**USP51**	CAUAGUGUUUCUACCACCA	SASI_Hs02_00372924	CAAAGCUACCAGGAGUCUA	SASI_Hs02_00372925
**USP52**	CUGUCUACCUGUUCCAUAU	SASI_Hs02_00329594	CAGUUUGACAUGAAUUGGA	SASI_Hs02_00329595
**USP53**	GAACCAAGUUUAGAAGUGA	SASI_Hs01_00234222	CAGCUAAGUUAAGUCACAU	SASI_Hs01_00234224
**USP54**	GACUUAGCAGAAGAUGUUA	SASI_Hs02_00366408	CCAAGUAUUGUUAAGCCAA	SASI_Hs02_00366409
**USP55**	CCAUAUUCAUGUUGCACUU	SASI_Hs02_00340306	GUAUCGAGCAAAUAAUCAU	SASI_Hs01_00128479
**CYLD**	GAACGAUGUAGAAUAUUAU	SASI_Hs02_00309208	GAACAGAUUCCACUCUUUA	SASI_Hs02_00309209
**DUB3**	CUAUCAUUGCGGUCUUUGU	SASI_Hs02_00372940	CAACAAACUUGCCAAGAAU	SASI_Hs02_00372941
**ATXN3**	GAAGAAUUAGCACAACUAA	SASI_Hs01_00043300	CAAAUGAUGGCUCAGGAAU	SASI_Hs01_00043301
**ATXN3L**	CAUUAUCAUCUAUGGGAUA	SASI_Hs02_00379672	CAAUUAACAUGGAUUUCAU	SASI_Hs02_00379673
**JOSD1**	CGGGAUACGCUGCAAGAGA	SASI_Hs02_00346483	CACUUCAGACCAAAGGCUA	SASI_Hs01_00072064
**JOSD2**	GGUGGACGGUGUCUACUAC	SASI_Hs01_00128488	GCAACUAUGAUGUCAAUGU	SASI_Hs01_00128489
**JOSD3**	GACAUCUGAUGCUGUGGAA	SASI_Hs01_00119698	GAUAAAUCAGGAAUAGAUU	SASI_Hs01_00119700
**TNFAIP2**	GGAAGAAAUACACAUAUUU	SASI_Hs01_00033556	GGAUGUUACCAGGACAUUU	SASI_Hs01_00033557
**OTUD7B**	CAACUAAUUGGCUCAUCAA	SASI_Hs01_00089029	CUGAUCAAGCUUGCCUCAA	SASI_Hs01_00089030
**OTUD7A**	GUCCUAGCCCAUAUAUUAA	SASI_Hs01_00055782	GACUUGAUCGAGCAGGCAA	SASI_Hs01_00055783
**OTUD4**	UAAUUUAUCGGGAACCAAA	SASI_Hs02_00323440	GUUUCUCCUUCACAAGUAA	SASI_Hs02_00323441
**PARP11**	CACAAUCAAACACAUGAAU	SASI_Hs01_00173656	GUGUUCAGUUAGCAGUGAA	SASI_Hs02_00353319
**OTUD6A**	CACAAUCAAACACAUGAAU	SASI_Hs01_00173656	GUGUUCAGUUAGCAGUGAA	SASI_Hs02_00353319

**YOD1**	GAAAUAUGUGUAGUGGAUA	SASI_Hs01_00052848	GUAACUUCCCUGAUCCAGA	SASI_Hs01_00052849
**OTUD6B**	GAAGUCAGACCGCUGAGUA	SASI_Hs01_00222466	CUACUAAGGAGAAUAAGAU	SASI_Hs02_00348712
**OTUD5**	CACUAUAAUUCAGUGGUGA	SASI_Hs01_00052828	GGACUUUACCACCUACAUU	SASI_Hs01_00052829
**OTUB1**	GCAAGUUCUUCGAGCACUU	SASI_Hs01_00080525	CCUGGAAGUUCCUUAGGGA	SASI_Hs02_00350395
**OTUB2**	CAGAGUGCCUCGGACCACA	SASI_Hs01_00010701	GACAUCAAAGACUUCUGCA	SASI_Hs01_00010702
**OTUD1**	CUGAUUUCUCACAGUGUAA	SASI_Hs01_00270248	GAUGUUUCAUGAUAGCUUU	SASI_Hs01_00270249
**OTUD3**	GAAAUCAGGGCUUAAAUGA	SASI_Hs02_00347304	GUAGUGAUUCAUCAACUUA	SASI_Hs02_00347305
**ZRANB1**	GAACUUGAAGUAGACUUUA	SASI_Hs01_00155779	GAAUCGUCCUUCUGCCUUU	SASI_Hs02_00350242
**VCPIP1**	GUAACUGCCUUUCAGGGAA	SASI_Hs02_00357704	GAUGUAUGGUCUUAUGCAA	SASI_Hs01_00165636
**STAMBP**	CCAAAGAAUAUACAGAAUA	SASI_Hs01_00182263	CAAAGUGCUGGGUCUGAUU	SASI_Hs01_00182264
**STAMBPL1**	GAGUUAGCCCGAGGUCAAA	SASI_Hs01_00241436	CAAUUCCUUGCUGAAUGUA	SASI_Hs01_00241437
**BRCC36**	GUCAGAAUUGUUCACAUUC	SASI_Hs01_00177040	CAUCUUACGACGUUCUGAU	SASI_Hs01_00177042
**COPS5**	CCAUUUGUAGCAGUGGUGA	SASI_Hs02_00342404	GUGAAUCUUGGCGCCUUUA	SASI_Hs01_00209042
**COPS6**	GAUCUUCCUGUCAGCGUUU	SASI_Hs02_00342398	CACACUGGUUGGUCAACUU	SASI_Hs01_00117938
**EIF3H**	CAAGGAUCUCUCUCACUAA	SASI_Hs01_00072290	CAAAUAUCACCUUUGAGUA	SASI_Hs01_00072291
**EIF3F**	GAGGAUGUACUGUCUGGAA	SASI_Hs01_00246686	CCGAUGACUUUGAGACCAU	SASI_Hs02_00336223
**IFP38**	CAUAGUGUUGCAAUAUGCA	SASI_Hs01_00017774	CAUAUGAGCAUCAAAGCCU	SASI_Hs01_00017775
**PSMD14**	GGUCUUAGGACAUGAACCA	SASI_Hs02_00340316	GUGAUUGAUGUGUUUGCUA	SASI_Hs01_00024446
**PRPF8**	CACGUAUCAAGAUUGGACU	SASI_Hs01_00015918	GGAUUAUGAUGCGCCGAGA	SASI_Hs01_00015919
**PSMD7**	CGUGUUGUUGGUGUGCUUU	SASI_Hs02_00334491	CACUUGUUACGAGAUAUCA	SASI_Hs02_00334492
**CEZANNE**	CAACUAAUUGGCUCAUCAA	SASI_Hs01_00089029	CUGAUCAAGCUUGCCUCAA	SASI_Hs01_00089030
**CEZANNE2**	GUCCUAGCCCAUAUAUUAA	SASI_Hs01_00055782	GACUUGAUCGAGCAGGCAA	SASI_Hs01_00055783
**MPND**	CCGAGAUGCUGCUGGUGGA	SASI_Hs01_00162447	GGAGUGAGGUCGUGGGUUA	SASI_Hs01_00162448
**MYSM1**	CUUACGAGAUAUUGACACA	SASI_Hs02_00317976	GACAAGAUGGAUAAUAGAA	SASI_Hs02_00317977
**SENP1**	GAAACAGCCGAAGUCUUUA	SASI_Hs01_00206194	GACAUUUGGACCGAUCUUU	SASI_Hs01_00206195
**SENP2**	GGAAUAAGUGACUAUCCAA	SASI_Hs01_00048880	GCUGUAACAGAGAUGAUUU	SASI_Hs02_00354976
**SENP3**	GUGCAUUGGUCCCUCAUCU	SASI_Hs01_00092168	CUGAUGCCAGCAUCCUCAU	SASI_Hs01_00092171
**SENP5**	CUGGUAACCAAAGGAUAUA	SASI_Hs02_00366591	GUGCUAAGACCAAGUUCAA	SASI_Hs01_00030279
**SENP6**	CAUACAGAUGGCUUAAGCA	SASI_Hs01_00034500	CCAUUAUUAAGAACGUCAA	SASI_Hs01_00034502
**SENP7**	CAAUAGCAGUGAUUGUGGA	SASI_Hs02_00312204	CAGUAUUAUGCCUAGUAAU	SASI_Hs02_00312205
**SENP8**	CAGUUCAUCAAGUGCACUA	SASI_Hs01_00195113	CCAACUUAUUUGAACAUUU	SASI_Hs01_00195114
**Control**	UGGUUUACAUGUCGACUAA	Human Non-Target 1_s	UGGUUUACAUGUUGUGUGA	Human Non-Target 2_s
**Control**	UGGUUUACAUGUUUUCUGA	Human Non-Target 3_s	UGGUUUACAUGUUUUCCUA	Human Non-Target 4_s

Supplementary Table 2: E2 ubiquitin-conjugaiting siRNA library

Target	siRNA_1	Name	siRNA_2	Name	siRNA_3	Name
**AKTIP**	GUUUACAUCCCUGAUAACU	SASI_Hs01_00086240	GAAUUUACCUUGGUUGUGA	SASI_Hs01_00086241	GCGUAUUUAAGUUUACAGU	SASI_Hs01_00086242
**BIRC6**	GUUAUGAGCUGCUUGUAGA	SASI_Hs01_00172666	GCUACAACAGGACUGACUA	SASI_Hs02_00349133	GGUACAAUCACAUCUAGCA	SASI_Hs02_00349134
**TSG101**	CUCAAUGCCUUGAAACGAA	SASI_Hs01_00180607	CUAUUGAAGACACUAUCUU	SASI_Hs01_00180608	CUUAUAGAGGUAAUACAUA	SASI_Hs02_00341284
**UBE2A**	CUUCUACCCUCUCCUUAAA	SASI_Hs01_00056615	GUCCUUCACUUGUGUUGGA	SASI_Hs02_00335488	GUACCUGGGUUACUUGCAA	SASI_Hs01_00056617
**UBE2B**	CUGGAUGAACCGAAUCCUA	SASI_Hs01_00017234	CUCCAUCUAUUGAUAGUUU	SASI_Hs01_00017235	CUCUCCAUCUAUUGAUAGU	SASI_Hs01_00017236
**UBE2C**	GCUCUCCAUCCAGAGCCUU	SASI_Hs01_00219473	CCAACAUUGAUAGUCCCUU	SASI_Hs01_00219474	GGACCAUUCUGCUCUCCAU	SASI_Hs01_00219475
**UBE2D1**	CCUUAAGACUGUGAUGAGA	SASI_Hs01_00156911	GAUAUUGCACAAAUCUAUA	SASI_Hs01_00156912	CAUAUCAAGGUGGAGUCUU	SASI_Hs01_00156913
**UBE2D2**	CUGAGAUUGCUCGGAUCUA	SASI_Hs01_00228722	GAAAUACUGUACUUCUGUA	SASI_Hs01_00228723	CCAUUUGACUGCUUUCUAU	SASI_Hs01_00228724
**UBE2D3**	GAGAAUUGGUAUUGAAUUU	SASI_Hs01_00192688	GCUUUAUGCUCAUAACUGA	SASI_Hs01_00192689	GGAGAAUUGGUAUUGAAUU	SASI_Hs01_00192690
**UBE2D4**	GCAGCAUGUUGGUGCCAUU	SASI_Hs01_00206454	CUCAUCUGCUGCAGAACCA	SASI_Hs01_00206455	GGCUUUGUUCGCACCUCAU	SASI_Hs01_00206457
**UBE2E1**	GCUUGUAGUCUGUAAAUUU	SASI_Hs01_00147122	GGUAUCUUGCUACAGUAGA	SASI_Hs01_00147123	CUGAAAUGUAGUACAGAAA	SASI_Hs01_00147124
**UBE2E2**	GAAUGGAGGUCAACUAUAU	SASI_Hs01_00102037	GGUUACCUUCCGAACAAGA	SASI_Hs01_00102038	CGGCUUUAACUAUUUCUAA	SASI_Hs01_00102039
**UBE2E3**	GUGUAUAUGUUAUACUGAU	SASI_Hs01_00107201	GUAGAUUAGUUAUGUUUAA	SASI_Hs01_00107202	CAUCUGUCUGGACAUCCUU	SASI_Hs01_00107203
**UBE2F**	GAGUUGUCUGCUUACCUUA	SASI_Hs01_00199216	GAGAGAACAUUCAAUUGAU	SASI_Hs01_00199220	CGGAAUAAAGUGGAUGACU	SASI_Hs02_00362455
**UBE2G1**	CAACAUGAUCCGGAUAAUA	SASI_Hs01_00219877	GGAAGAAUUGUAAAGACCU	SASI_Hs01_00219879	CACCUAUUGCUGGACUUCU	SASI_Hs02_00335492
**UBE2G2**	CACCCUUUCUGGGUCCUUC	SASI_Hs01_00077052	CCAGAACGCUCCUUCUGAA	SASI_Hs01_00077053	CCCAGAACGCUCCUUCUGA	SASI_Hs01_00077054
**UBE2H**	CAGCUCUUGGAUGUGAAGA	SASI_Hs02_00335498	GACUUAAUGAAUUUGUAGU	SASI_Hs01_00024164	GAGAGUAAACAUGAGGUUA	SASI_Hs01_00024165
**UBE2J1**	GAAGUCAAUUCAUCUGGAA	SASI_Hs01_00027696	CCUUUAGAGGAUAACCUUU	SASI_Hs01_00027697	CUUAUGAGCUGUGACUCAA	SASI_Hs01_00027698
**UBE2J2**	GGUUUGCAGCCUUUGCUUA	SASI_Hs01_00095058	GAGACGUCGGACUUCACGA	SASI_Hs01_00095059	CUGAAGUCGUGGAGGAGAU	SASI_Hs01_00095060
**UBE2K**	GCAUAGAGAGCUGCUGAUA	SASI_Hs02_00325256	GGAAGAAACAUGCUACUUU	SASI_Hs02_00325257	GUAGAUCUUGUAGAUGAGA	SASI_Hs02_00325258
**UBE2L3**	CUAGUUCUGCUCUCUUUGU	SASI_Hs01_00049984	GGCUUAUUGUUCCUGACAA	SASI_Hs01_00049991	CUGACAACCCUCCAUAUGA	SASI_Hs01_00049985
**UBE2L6**	GAUUUAGGUGCUAAACCGU	SASI_Hs01_00205565	CACUGAUGGGAAUAUACAG	SASI_Hs01_00205566	CCCAUCAUCAGCAGUGAGA	SASI_Hs01_00205567
**UBE2M**	CCUGCAGUAUCUCUUCUUG	SASI_Hs01_00232487	CUCCAUAAUUUAUGGCCUG	SASI_Hs01_00232488	GGUGAAGUGUGAGACAAUG	SASI_Hs01_00232489
**UBE2N**	CAGACAUCUUCAGUCCUUU	SASI_Hs01_00012964	GAAGAAUAUGUUUAGAUAU	SASI_Hs01_00012965	CCUCUUUGUUUGCAUUUAA	SASI_Hs01_00012966
**UBE2NL**	GUAUUUAAAUUGAUCCAAU	SASI_Hs01_00199377	CCAAUCAUUAAGUGUGCAU	SASI_Hs01_00199378	CCAGACAUCUUCAGUUAUU	SASI_Hs01_00199379
**UBE2O**	GAGACUUCGUGGUAGAUAA	SASI_Hs01_00037229	CUGACAUCGUCAUCCGCAU	SASI_Hs01_00037230	CAUGGAUUACCAAGAGUUU	SASI_Hs01_00037231
**UBE2Q1**	CUGUAUGAUUGGAAUGUCA	SASI_Hs01_00241781	GUAUGAUUGGAAUGUCAAA	SASI_Hs01_00241782	CAUGGAACUUCUCACCAAA	SASI_Hs01_00241783
**UBE2Q2**	GGCUAAAUAUGUUGACUGU	SASI_Hs01_00128047	GGUGUUACCUGUUCUCUCA	SASI_Hs01_00128048	CUGUUGUAUGUUUGGACUA	SASI_Hs01_00128049
**UBE2R1**	CUCCUUAGACGACAGACUA	SASI_Hs02_00337425	GGACCAUUCUCCUGAGUGU	SASI_Hs01_00071093	GAAGGAUCGGGAGUACACA	SASI_Hs01_00071094
**UBE2R2**	GAGUAACCCUCCACAGAAU	SASI_Hs01_00058641	CUUCAGAUUCUUGACCAAA	SASI_Hs01_00058647	CACAGAAUGUCUGAAUUCU	SASI_Hs01_00058642
**UBE2S**	CCCGUCUGCUCACAGAGAU	SASI_Hs01_00070712	GCCUGCUCUUGGAGAACUA	SASI_Hs01_00070713	CGCCUGCUCUUGGAGAACU	SASI_Hs01_00070714
**UBE2T**	GCCAACACACCUUAUGAGA	SASI_Hs01_00129086	CUGCUCAUGUCAGAACCCA	SASI_Hs01_00129087	CCAACACACCUUAUGAGAA	SASI_Hs01_00129088
**UBE2U**	CUGCUAAGCCUGUAAGUGA	SASI_Hs01_00182543	CCAGAAUACUGGUUAAAGA	SASI_Hs01_00182544	GAAUACUGGUUAAAGAUGA	SASI_Hs02_00366264
**UBE2V1**	GCCUAAUGAUGUCUAAAGA	SASI_Hs01_00020112	CUAUUUAAAUUGAUUCCCA	SASI_Hs01_00020119	CCACUGUCCACGUAGUUGA	SASI_Hs01_00020121
**UBE2V2**	GUUGUACUUCAAGAGCUAA	SASI_Hs01_00031079	GUCAGUUAGAUUUGUAACA	SASI_Hs01_00031080	CUGACUACUAGUAGUAUCA	SASI_Hs01_00031082
**UBE2W**	CAUGUAACAAGAAUCCAAA	SASI_Hs02_00303787	CAAUCAGUUUGUCUUAGCA	SASI_Hs02_00303788	CAACUGUUUAUAUUUGGAU	SASI_Hs01_00231191
**UBE2Z**	CUGUUGACAUGACUAAGAU	SASI_Hs01_00048090	GAAUGUAUCCGGCACGAGA	SASI_Hs01_00048091	GAAAGUCUGCUUGAGUAUU	SASI_Hs01_00048092
**UEV3**	GAUGUAUCAGGGUAAUACA	SASI_Hs01_00049331	GUAUCAGGGUAAUACAUAU	SASI_Hs01_00049334	CCAAUUCGUUUCUGGAUUU	SASI_Hs01_00049335

Supplementary Table 3: Mesothelioma IHC analysis

	Specimen ID	Histology	UBE2E3	UBE2E1	UBE2E2	ASXL2
WT BAP1	MVB0020	epithelial	positive on normal meso cells	Negative on tumor cells, positive mostly nuclear blood vessels, muscles cells and lymphocytes	Positive in cytoplasm of tumor cells. Stains cytoplasm of inflammatory cells, muscle cells and blood vessels.	Focally Positive in cytoplasm of tumor cells. Stains cytoplasm of blood vessles and inflammatory cells.
	MVB0061	sarcomatoid	mostly positive on nuclear and cytoplasmic, area where tumor cells are negative	n/a damaged sample	Negative in tumor cells	Negative
	MVB0077	biphasic	positive, mostly cytoplasmic	Negative in tumor cells	Strongly positive in cytoplasm of tumor cells	Focally positive in cytoplasm of tumor cells.
	MVB0428	epithelial	focally positive on both nuclear and cytoplasmic	Negative in tumor cells	Positive cytoplasmic staining in tumor cells *	Positive in nucleus of tumor cells*
	MVB0436	epithelial	positive, mostly nuclear	Negative in tumor cells	Positive cytoplasmic in tumor cells	Focally positive in cytoplasm of tumor cells.
	MVB0453	high grade epithelial/sarc	positive, mostly nuclear	Negative in tumor cells	Positive cytoplasmic in tumor cells * pic of blood vessels	Positive in cytoplasm only *
	MVB0456	epithelial/biphasic	positive, nuclear	Negative in tumor cells	Positive in cytoplasm of tumor cells	Focally positive mostly in cytoplasm in tumor cells. In normal mesothelial cells, focally positive 40%
	MVB0469	biphasic	positive, mostly nuclear	Focally positive nucleus 30% in tumor cells, Positive in lymphocytes nucleus	Positive in cytoplasm of tumor cells 70%	Positive nuclear and cytoplasmic staining
	MVB0479	biphasic	positive, nuclear	Focally positive in nucleus of tumor cells 30%	Negative in tumor cells	Negative
	MVB0489	epithelial	positive, nuclear	Negative in tumor cells	Negative in tumor cells	Negative
BAP1 mutant	MVB0026	epithelial	positive, mostly nuclear	Focally positive nuclear 30% of tumor cells, Some tumor cells also have nuclear plus cytoplasmic staining. Nuclear staining is always much stronger than cyto. Negative tumor cells are negative in cyto and nucleus. Stains nuclei of lymphocytes	Positive Largely cytoplasmic staining in tumor cells.	Focally positive nuclear 30% tumor cells and weak cytoplasmic staining.
	MVB0029	epithelial	focally positive, weak mostly cytoplasmic	Negative in tumor cells. Focally, some tumor cells show cytoplasmic staining. lymphocytes show nuclear staining	focally, 5%of tumor cells show cytoplasmic staining, tumor cells largely negative	Negative
	MVB0389	epithelial	negative, mostly. Few areas of positivity	Negative in tumor cells	Focally positive in cytoplasm of tumor cells 20% *	Negative
	MVB0405	epithelial	negative, largely. Some nuclear, focal positivity	Negative in tumor cells with some focal rare cytoplasmic staining. Lymphocytes and blood vessels are positive in nuclei	Focally positive in cytoplasm 10% of tumor cells. Normal mesothelial cells positive in cytoplasm	Negative on tumor cells, shows some nuclear positivity on normal mesothelial
	MVB0409	epithelial	negative, mostly. Some weak focal nuclear staining	Negative in tumor cells	Focally positive in cytoplasm 20% of tumor cells	Positive in nucleus of tumor cells
	MVB0432	epithelial	negative. Some weak focal cytoplasmic staining	Negative in tumor cells	Focally Positive in cytoplasm of tumor cells 70%	Negative
	MVB0447	epithelial	negative	Negative in tumor cells	Negative in tumor cells	Negative
	MVB0451	epithelial	positive, nuclear	Focally positive in nucleus of tumor cells 10%	Focally positive in cytoplasm *	Negative
	MVB0471	epithelial	negative. Occassional weak focal cytoplasmic staining	Focally positive in nuclei of tumor cells 50%	Focally positive in cytoplasm of tumor cells 10%. Plasma cells positive in cytoplasm	Positive in nucleus of tumor cells 50%
	MVB0491	biphasic	focally positive ~50% in nucleus	Positive in nucleus and cytoplasm 50% of tumor cells	Positive in cytoplasm of tumor cells	Positive in nucleus and cytoplams of tumor cells 20%

Supplementary Table 4: List of Oligonucleotides used

Primer name	Sequence
**ASXL2ΔASXN_Forward**	GGCCGCACATATGGGGAAGAAGTCATTCCAGA
**ASXL2ΔASXN_Reverse**	GATCTCTGGAATGACTTCTTCCCCATATGTGC
**ASXL2ΔPHD_Forward**	GGCTCCGCGGCCGCCCCCTTCACCATGAGGGAAAAGGGACGTAGGAAG
**ASXL2ΔPHD_Reverse**	GCCTTACCTGGCGCGCCTCATACCTG GCTACCTGGTACAGCAGAGTT
**DEUBAD(ASXL2) K289R_Forward**	GCACTGATCAACAGGCACACATTTTCAGTCCTT
**DEUBAD(ASXL2) K289R_Reverse**	GAAAATGTGTGCCTGTTGATCAGTGCTCGCAGAT
**DEUBAD(ASXL2) K340R_Forward**	CCAAGGCTGGAGGGAAAGACTCTCAGAAGGTGA
**DEUBAD(ASXL2) K340R_Reverse**	AGAGTCTTTCCCTCCAGCCTTGGGCTGCTGAAG
**DEUBAD(ASXL2) K365R_Forward**	GAAGGAGAAAAGAGTGGAGCCATGGAAAGAACA
**DEUBAD(ASXL2) K365R_Reverse**	CATGGCTCCACTCTTTTCTCCTTCTCAATCTCTTG
**DEUBAD(ASXL2) K370R_Forward**	GGAGCCATGGAGAGAACAATTCTTTGAAAGCTAC
**DEUBAD(ASXL2) K370R_Reverse**	AGAATTGTTCTCTCCATGGCTCCACTTTTTTCTCC
**DEUBAD(ASXL2) K390R_Forward**	TGAAGATTCTAGGAAATTGACAGCTTCTCCCAGT
**DEUBAD(ASXL2) K390R_Reverse**	AGCTGTCAATTTCCTAGAATCTTCAAGGCTCAGGC
**DEUBAD(ASXL2) K391R_Forward**	GAAGATTCTAAGAGATTGACAGCTTCTCCCAGTGA
**DEUBAD(ASXL2) K391R_Reverse**	AGAAGCTGTCAATCTCTTAGAATCTTCAAGGCTC
**DEUBAD(ASXL1) K351R_Forward**	GGAACAATGGAGAGAAAAGTTCTTTGAAGACTAC
**DEUBAD(ASXL1) K351R_Reverse**	AGAACTTTTCTCTCCATTGTTCCACCTTCTTTTC
**DEUBAD(ASXL1) K353R_Forward**	CAATGGAAAGAAAGGTTCTTTGAAGACTACTATGG
**DEUBAD(ASXL1) K353R_Reverse**	CTTCAAAGAACCTTTCTTTCCATTGTTCCACCT
**DEUBAD(ASXL1) K362R_Forward**	CTATGGACAGAGGCTGGGTTTGACCAAAGAAGAG
**DEUBAD(ASXL1) K362R_Reverse**	GTCAAACCCAGCCTCTGTCCATAGTAGTCTTCAA
**GFP_gRNA_Forward**	caccgGGGCGAGGAGCTGTTCACCG
**GFP_gRNA_Reverse**	aaacCGGTGAACAGCTCCTCGCCCc
**hUBE2E3_gRNA-1_Forward**	caccgCTGGAGCGGCTGGGTCTCGC
**hUBE2E3_gRNA-1_Reverse**	aaacGCGAGACCCAGCCGCTCCAGc
**hUBE2E3_gRNA-2_Forward**	caccgGGTGCTGGGGCTCTCATCAT
**hUBE2E3_gRNA-2_Reverse**	aaacATGATGAGAGCCCCAGCACCc
**dsRNAi_UbcD2_Forward(+T7)**	taatacgactcactatagggTTCGCTTAGCAACCGAAAAC
**dsRNAi_UbcD2_Reverse(+T7)**	taatacgactcactatagggCGCATTACGCATTAAAATAGGC
**dsRNAi_Calypso_Forward(+T7)**	taatacgactcactatagggATGCCTTGTTCCACACATCC
**dsRNAi_Calypso_Reverse(+T7)**	taatacgactcactatagggGAAATGGCCTCCTCATCCTT
**dsRNAi_Luc_Forward(+T7)**	taatacgactcactatagggTGCTGGACAGCTTCATCAAC
**dsRNAi_Luc_Reverse(+T7)**	taatacgactcactatagggATGTCCTCCTCGATGTCTGG
**Asx_K325R_Forward**	GGATCCCTGGcgcCTGAAACACTTTGAGCCCTT
**Asx_K325R_Reverse**	AGTGTTTCAGgcgCCAGGGATCCAACTTGTTCT
**DEUBAD(Asx)_Forward**	GCGGCCGCcATGAGCGTTAAACCGCGT
**DEUBAD(Asx)_Reverse**	GGCGCGCCCCTGCAGGTTAATTTTTACA
**Flag_Calypso_Forward**	actcGGATCCatgGACTACAAAGACGATGACGACAAGAACGCTGCTGGAGGAGGTAGTG
**Flag_Calypso_Reverse**	gagtGATATCggcgcgccCTACTTCCGTTTTCTGCACTTGTT

Supplementary Table 5: List of antibodies used

Antibody	Source	Catalogue number	Dilution
**Mouse monoclonal anti-BAP1 (C4)**	Santa Cruz	sc-28383	1:1000
**Rabbit polyclonal anti-BAP1 (H300)**	Santa Cruz	sc-28236	1:1000
**Rabbit polyclonal anti-YY1 (H414)**	Santa Cruz	sc-1703	1:1000
**Mouse monoclonal anti-CDC6 (180)**	Santa Cruz	sc-9964	1:1000
**Mouse monoclonal anti-Tubulin (B-5-1-2)**	Santa Cruz	sc-SC-23948	1:2000
**Rabbit polyclonal anti-ASXL2**	Bethyl Laboratories	A302-037A	1:2000
**Mouse monoclonal anti-Flag (M2)**	Sigma-Aldrich	F3165	1:2000
**Rabbit polyclonal anti-GST**	Sigma-Aldrich	G7781	1:20000
**Mouse monoclonal anti-MYC**	Covance	9E10	1:1000
**Rabbit polyclonal anti-H2Aub K119**	Cell signaling	D27C4	1:30000
**Mouse monoclonal anti-MBP**	Cell signaling	E8038	1:5000
**Mouse anti-V5**	Abcam	ab27671	1:2000
**Mouse anti-Myc (home-made)**	Mashtalir et al., 2014	N/A	1:1000
**Mouse anti-HA (home-made)**	Mashtalir et al., 2014	N/A	1:1000
**Rabbit polyclonal anti-UBE2E1**	Boston Biochem	A-630	1:1000
**Rabbit polyclonal anti-UBE2E2**	Bethyl Laboratories	A303-485A	1:2000
**Mouse polyclonal anti-UBE2E3**	Abcam	ab66257	1:2000
**Mouse anti-Ubx**	DSHB	FP3.38	1:200
**Rabbit polyclonal anti-UBE2E1/UbcH6 (for IHC)**	Novus Biologicals	NBP1-78983	1:600
**Mouse monoclonal anti-UBE2E3 (7E8) (for IHC)**	Novus Biologicals	NBP2-03819	1:200
**Rabbit polyclonal anti-H3**	Abcam	ab1791	1:50000
**Rabbit polyclonal anti-UBE2O**	Novus Biologicals	NBP1-32020	1:2000
**Anti-mouse Alexa Fluor® 594**	Life Technologies	A11005	1:1000
**anti-mouse Alexa Fluor® 488**	Life Technologies	A11029	1:1000
**Anti-rabbit Alexa Fluor® 488**	Life Technologies	A11008	1:1000
**anti-rabbit Alexa Fluor® 594**	Life Technologies	A11012	1:1000

Supplementary Table 6: List of commercial enzymes used for ubiquitination assays

Recombinant Enzyme	Source	Catalogue number
**UBE1**	Boston Biochem	E-305
**UbcH5a/UBE2D1**	Boston Biochem	E2-616
**UbcH5b/UBE2D2**	Boston Biochem	E2-622
**His6-UbcH6/UBE2E1**	Boston Biochem	E2-630
**UbcH7/UBE2L3**	Boston Biochem	E2-640
**UbcH8/UBE2L6**	Boston Biochem	E2-644
**His6-UBE2E3**	Boston Biochem	E2-678
**His6-UbcH10/UBE2C**	Boston Biochem	E2-650
**His6-UBE2W Isoform 1**	Boston Biochem	E2-740
**His6-UBE2S**	Boston Biochem	E2-690
**His6-UBE2F**	Boston Biochem	E2-690
**UBE2K/E2-25K**	Boston Biochem	E2-603
**UBE2M/Ubc12**	Boston Biochem	E2-656
**His6-UbcH3/Cdc34**	Boston Biochem	E2-610
**His6-UBE2T**	Boston Biochem	E2-695
**His6-UBE2N/Ubc13**	Boston Biochem	E2-660
**His6-UBE2N (Ubc13)/UBE2V1 (Uev1) Complex**	Boston Biochem	E2-664
**His6 UBE2N/UBE2V2 Complex**	Boston Biochem	E2-666
**UbcH2/UBE2H**	Boston Biochem	E2-607
**His6-HR6A/UBE2A**	Boston Biochem	E2-612
**His6-HR6B/UBE2B**	Boston Biochem	E2-613
**UBE2I (Ubc9)**	Boston Biochem	E2-645
**His6-Uev1a/UBE2V1**	Boston Biochem	E2-662
**His6-Use1/UBE2Z**	Boston Biochem	E2-677
**His6-UBE2G2**	Boston Biochem	E2-680
**His6-UBE2G1/E2-17K**	Boston Biochem	E2-700
**His6-UBE2D4 (UbcH5D)**	Boston Biochem	E2-705
**His6-UBE2J2**	Boston Biochem	E2-710
**His6-UBE2R2/CDC34B**	Boston Biochem	E2-715
**His6-UBE2Q1-2**	Boston Biochem	E2-720
**His6-UBE2Q2-1**	Boston Biochem	E2-745
**His6-UBE2J1**	Boston Biochem	E2-750

Supplementary Table 7: Characteristics of the human patient's samples used in the study

ID numbers	Age	Gender	Genotype (WT or BAP1 mutant)	MM Diagnosis (Primary vs. Metastatic)	Type of Therapy
**MVB0020**	31	Female	WT	Primary and metastatic	Surgery and chemotherapy
**MVB0061**	78	Male	WT	Primary	Surgery
**MVB0077**	84	Male	WT	Primary	Talc Pleurodesis
**MVB0428**	66	Female	WT	Primary	Surgery and chemotherapy
**MVB0436**	32	Male	WT	Primary	Surgery and chemotherapy
**MVB0453**	34	Female	WT	Primary	Surgery and chemotherapy
**MVB0456**	23	Female	WT	Primary	Surgery
**MVB0469**	46	Female	WT	Primary	Surgery and chemotherapy
**MVB0479**	73	Male	WT	Primary	Surgery and chemotherapy
**MVB0489**	43	Male	WT	Primary	Surgery and chemotherapy
**MVB0026**	85	Male	BAP1 mutant	Primary	Talc Pleurodesis and radiation
**MVB0029**	73	Male	BAP1 mutant	Primary and metastatic	Surgery
**MVB0389**	60	Male	BAP1 mutant	Primary	Surgery and chemotherapy
**MVB0405**	67	Male	BAP1 mutant	Primary	Surgery and radiation
**MVB0409**	63	Male	BAP1 mutant	Primary	Surgery and chemotherapy
**MVB0432**	77	Male	BAP1 mutant	Primary	Chemotherapy
**MVB0447**	51	Female	BAP1 mutant	Primary and metastatic	Surgery and chemotherapy
**MVB0451**	71	Male	BAP1 mutant	Primary	Chemotherapy
**MVB0471**	59	Male	BAP1 mutant	Primary and metastatic	Surgery and chemotherapy
**MVB0491**	68	Female	BAP1 mutant	Primary	Surgery, chemotherapy and talc pleurodesis
